# Well‐Defined Poly(Ester Amide)‐Based Homo‐ and Block Copolymers by One‐Pot Organocatalytic Anionic Ring‐Opening Copolymerization of *N*‐Sulfonyl Aziridines and Cyclic Anhydrides

**DOI:** 10.1002/anie.202015339

**Published:** 2021-02-24

**Authors:** Jiaxi Xu, Nikos Hadjichristidis

**Affiliations:** ^1^ King Abdullah University of Science and Technology (KAUST) Physical Sciences and Engineering Division KAUST Catalysis Center Polymer Synthesis Laboratory Thuwal 23955 Saudi Arabia

**Keywords:** anhydrides, anionic copolymerization, *N*-sulfonyl aziridines, phosphazenes, poly(ester amide)s

## Abstract

We report a new synthetic methodology for poly(ester amide)s by anionic ring‐opening copolymerization of *N*‐sulfonyl aziridines and cyclic anhydrides. Phosphazenes organocatalysts have been found to promote a highly‐active, controlled, and selective alternating copolymerization in the absence of any competitive side reaction (zwitterionic mechanism and exchange transacylations). Mechanistic studies have shown first‐order dependence of the copolymerization rate in *N*‐sulfonyl aziridines and phosphazenes, and zero‐order in cyclic anhydrides. This one‐pot methodology leads not only to homopolymers but also to poly(ester amide)‐based block copolymers. Two catalytic cycles involving ring‐opening alternating copolymerization of *N*‐sulfonyl aziridines with cyclic anhydrides and ring‐opening polymerization of *N*‐sulfonyl aziridines have been proposed to explain the one pot synthesis of poly(ester amide)‐based homo‐ and block copolymers.

## Graphical abstract

Phosphazene organocatalysts have been found to promote a highly‐active, controlled, and selective alternating ring‐opening copolymerization of *N*‐sulfonyl aziridines and cyclic anhydrides. This one‐pot methodology leads not only to homopolymers but also to poly(ester amide)‐based block copolymers.

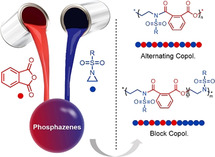

Poly(ester amide)s have gained an increasing interest in recent years as they combine, in the same macromolecule, the biodegradability/biocompatibility of polyesters with excellent thermal and mechanical properties of polyamides. Therefore, they are widely used in a variety of fields, including drug delivery, hydrogels, non‐viral gene carriers, composite materials, and tissue engineering.^[1]^ Poly(ester amide)s are traditionally produced by polycondensation reactions from ester‐containing diamines with dicarboxylic acids or their derivatives. However, this yields by‐products and low molecular weight polymers with broad distributions. In contrast to polycondensation, ring‐opening polymerization (ROP) of morpholino‐2,5‐diones is more atom‐economical.^[2]^ Furthermore, the ROP of morpholino‐2,5‐diones is generally catalyzed by metals or enzymes in bulk at high temperatures, resulting in uncontrolled products due to unavoidable side reactions;^[3]^ a significant amount of organocatalysts is required to suppress side effects.^[4]^ The ring‐opening copolymerization of lactams and lactones can also produce copolymers with ester and amide linkages, although not alternating and contaminated by exchange transacylation reactions products.^[5]^

Inspired by the rapid advancement in polyester synthesis by ring‐opening alternating copolymerization (ROAP) of epoxides with cyclic anhydrides,^[6]^ we thought that the ROAP of aziridines with cyclic anhydrides is a potential synthetic methodology for poly(ester amide)s. In addition, the functionalization of aziridines ring‐nitrogen will increase the potential structural diversity of poly(ester amide)s.

Already, a limited number of reports are available for copolymerization of aziridines with cyclic anhydrides.^[7]^ Although aziridines have a similar chemical structure and ring strain to epoxides, the strong nucleophilic ring‐nitrogen allows aziridines to act as nucleophilic monomers, in contrast to the electrophilic epoxide monomers. Aziridines, even without initiators, copolymerize with cyclic anhydrides through a zwitterionic mechanism, leading to insoluble oligomers and copolymers with uncontrolled molecular weight.^[8]^ Less nucleophilic *N*‐alkyl aziridines were designed to control their copolymerization behavior,^[9]^ but, unfortunately, copolymers with uncontrolled molecular weight and composition were obtained. Even the use of benzyl alcohol as an initiator for the copolymerization of *N*‐alkyl aziridines with cyclic anhydrides has led to uncontrolled products due to the unavoidable zwitterionic side reactions.^[10]^ Several competitive side reactions have also been observed, such as intra‐ and intermolecular exchange transacylations. To the best of our knowledge, there is no report on the controlled and selective copolymerization to produce poly(ester amide)s from aziridine derivatives with cyclic anhydrides.

Since the first report in 2005 by Bergman and Toste,^[12]^ among aziridines, the *N*‐sulfonyl aziridines have been the most suitable monomers for anionic ROP. The strong electron‐withdrawing sulfonyl group weakens the nucleophilicity of the ring‐nitrogen and thus making the *N*‐sulfonyl aziridines electrophilic monomers. To date, however, only racemic *N*‐sulfonyl aziridines have been successfully polymerized to produce linear polymers.^[11]^ The polymerization of unsubstituted or stereoregular *N*‐sulfonyl aziridines is challenging as they produce only insoluble oligomers. The copolymerization of two ring‐unsubstituted *N*‐sulfonyl aziridines [*N*‐(methylsulfonyl)aziridine and *N*‐(*sec*‐butylsulfonyl)aziridine], produces soluble random copolymers.^[12]^ Copolymerization of unsubstituted *N*‐sulfonyl aziridines with cyclic anhydrides was a possible method to solve the solubility problem, but copolymerization with cyclic anhydrides was challenging, as unsubstituted *N*‐sulfonyl aziridines are easily undergone homopolymerization.

Our first attempt was to copolymerize *N*‐tosylaziridine (TAz) with phthalic anhydride (PA) in the absence of an initiator and a catalyst (Entry 1, Table 1). There was no peaks of copolymers in the ^1^H NMR spectrum, indicating the absence of a spontaneous zwitterionic copolymerization (Scheme S1, a). The ring‐nitrogen in TAz does not show a nucleophilic character because the strong electron‐withdrawing toluene sulfonyl group delocalizes the lone pair electrons. Even the addition of an initiator [BnN(H)Ts] in the mixture of TAz and PA did not promote copolymerization (Entry 2, Table 1), indicating that the nucleophilicity of an initiator is insufficient, and the presence of a catalyst is necessary.

**Table 1:** Copolymerizations of *N*‐sulfonyl aziridines with PA.^[a]^

Entry	Monom.(M)	Base	[TAz]_0_/[PA]_0_/[I]_0_/[Base]_0_	*T* [°C]	*t* [h]	Conv.^[b]^ [%]	*M*_n,theor_^[c]^ [kg mol^−1^]	*M*_n,NMR_^[b]^ [kg mol^−1^]	*Đ* ^[d]^ *M*_w_/*M*_n_
1	TAz	–	15/15/0/0	25	24	–	–	–	–
2	TAz	–	15/15/ 1/ 0	25	24	–	–	–	–
3	TAz	*t*‐BuP_4_	15/15/1/0.3	25	20	96	5.23	5.35	1.06
4					48	99	5.39	5.57	1.06
5	TAz	*t*‐BuP_2_	15/15/1/0.3	25	24	95	5.18	5.38	1.09
6					48	99	5.39	5.50	1.08
7	TAz	*t*‐BuP_1_	15/15/1/0.3	25	24	59	3.32	3.48	1.10
8					48	92	5.03	5.10	1.09
9	TAz	*t*‐BuP_4_	15/15/1/0.3	50	1	92	5.03	5.13	1.07
10					24	99	5.39	5.58	1.07
11	TAz	*t*‐BuP_4_	50/50/1/0.3	50	2.5	96	16.8	17.2	1.04
12	TAz	*t*‐BuP_4_	100/100/1/0.3	50	12	98	34.1	35.7	1.02
13	TAz	*t*‐BuP_2_	40/15/1/0.3	25	3	99^[e]^	5.39^[f]^	5.56	1.07
14^[g]^	TAz	*t*‐BuP_2_	15/15/1/0.3	25	24	95	5.04	5.12	1.07
15^[h]^	TAz	*t*‐BuP_2_	15/15/1/0.3	25	24	94	4.98	5.02	1.07
16^[i]^	TAz	*t*‐BuP_2_	15/15/1/0.6	25	24	99	5.27	5.31	1.08
17^[j]^	TAz	*t*‐BuP_2_	15/15/1/0.9	25	24	99	5.30	5.39	1.07
18^[k]^	TAz	*t*‐BuP_2_	15/15/1/0.3	25	24	99	5.39	5.41	1.07
19^[l]^	TAz	*t*‐BuP_2_	15/15/1/0.3	25	24	62	3.47	3.31	1.12
20	BAz	*t*‐BuP_4_	15/15/1/0.3	25	20	99	6.35	5.95	1.07
21	NAz	*t*‐BuP_4_	15/15/1/0.3	25	20	N.A.^[m]^	N.A.^[m]^	N.A.^[m]^	N.A.^[m]^

[a] The copolymerizations were initiated by BnN(H)Ts at 25 °C in THF ([PA]_0_=1.0 M). [b] TAz conversion was determined by ^1^H NMR in CDCl_3_ using integrals of the characteristic signals. [c] Calculated as follows: ([PA]_0_/[I]_0_) × conv. of TAz × (M.W. of *N*‐sulfonyl aziridines + M.W. of PA) + (M.W. of Initiator). [d] Determined by SEC traces at 35 °C in THF (1.0 mL min^−1^) using PSt standards. [e] PA conversion was determined by in situ FTIR. [f] Calculated as follows: ([PA]_0_/[I]_0_) × conv. of PA × (M.W. of *N*‐sulfonyl aziridines + M.W. of PA) + (M.W. of Initiator). [g] Initiated by benzoic acid. [h] Initiated by benzyl alcohol. [i] Initiated by 1,4‐benzenedimethanol. [j] Initiated by 1,3,5‐benzenetrimethanol. [k] In DMF. [l] In CH_2_Cl_2_. [m] Copolymers dissolved in solvent.

Phosphazene bases are excellent organocatalysts for ROP and ROAP because they combine high/tunable basicity and non‐nucleophilic nature.^[13]^ Although phosphazene bases exhibit high catalytic activity in ROP of lactones and ROAP of epoxides with cyclic anhydrides, the molecular weight distributions of the resultant polymers are broad because of the lability of esters under the polymerization conditions, when intermolecular transesterification and intramolecular back‐biting reactions are inevitable.^[14]^ Compared to the high catalytic efficiency of strong basic *t*‐BuP_4_, the weaker bases *t*‐BuP_1_ and *t*‐BuP_2_ are more suitable for the polymerization of monomers with ester groups.^[14]^ Unfortunately, the highly reactive *t*‐BuP_4_ causes more side reactions, and the well‐controlled/well‐selective *t*‐BuP_1_ and *t*‐BuP_2_ are limited to catalytic reactivity in the polymerization of ester‐containing monomers.

We tried to add *t*‐BuP_4_ into the mixture of TAz and PA in the presence of an initiator (Scheme 1 and Entry 3, Table 1). The copolymerization of TAz with PA reached 96 % conversion in 20 h. The molecular weight distribution of the resulting copolymers was surprisingly narrow (*Ð*=1.06, Figure 1 a, bottom). After prolonging the reaction time to 48 h (Entry 4, Table 1), the distribution remained the same (*Ð*=1.06, Figure 1 a, top), indicating the absence of competitive inter‐ and intramolecular exchange transacylations (Scheme S1, b). The produced negative charge on nitrogen, at the chain‐end, can be delocalized by the strong electron‐withdrawing toluene sulfonyl groups, revealing a low nucleophilic character; and thus, competitive transacylation exchanges are suppressed. This is the first time a super‐strong base, *t*‐BuP_4_, has been used to promote a high‐reactive, high‐controlled, and high‐selective organocatalytic polymerization of ester‐containing monomers.

**Figure 1:**
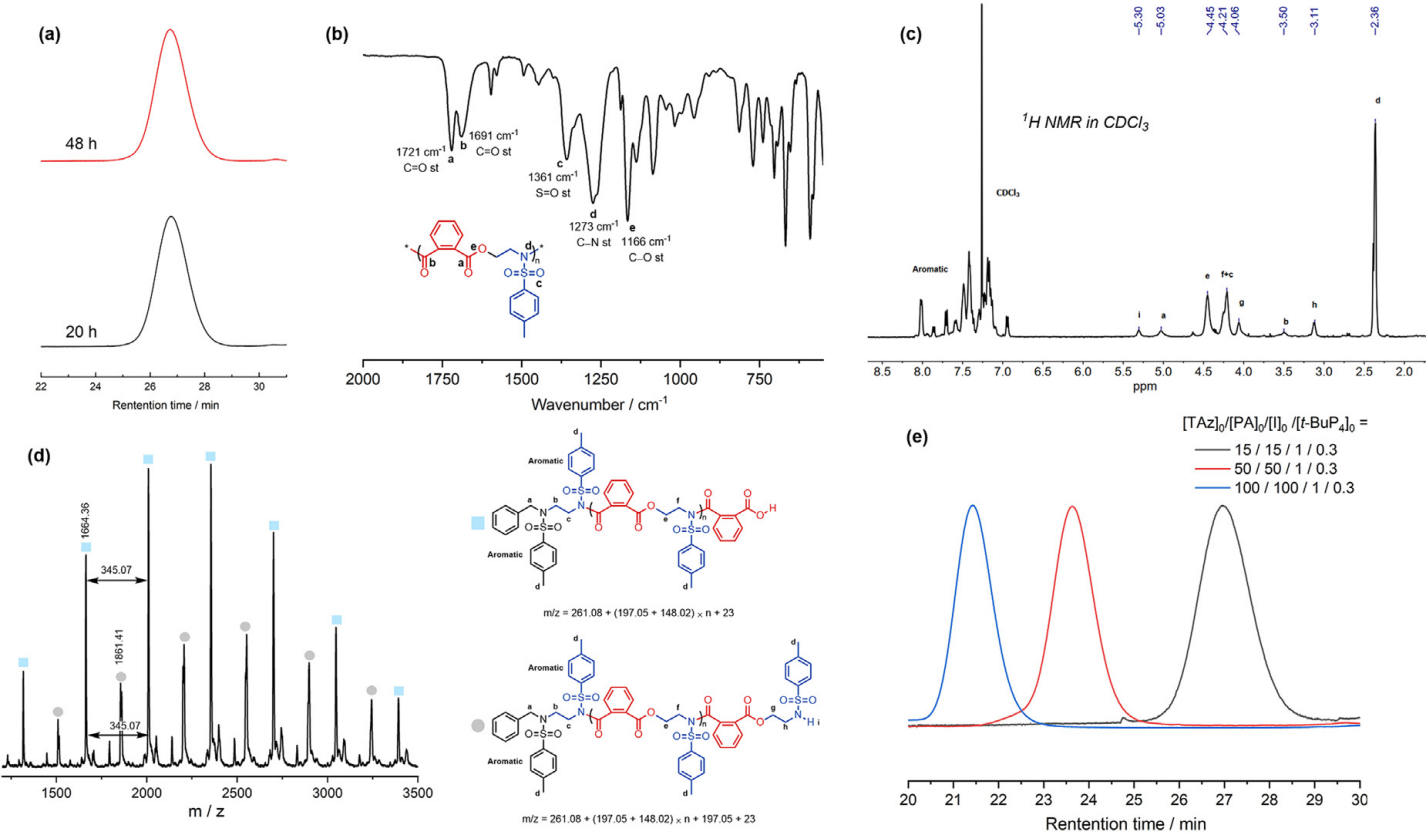
a) Bottom: SEC traces of copolymers of TAz and PA in 20 h (Entry 3, Table 1); Top: the reaction was prolonged to 48 h (Entry 4, Table 1); b) FTIR spectrum of the copolymers powder; c) ^1^H NMR spectrum (400 MHz, CDCl_3_, 25 °C) of copolymers. d) MALDI‐ToF MS spectrum of the copolymers. e) SEC traces of copolymers with different molecular weights (Entry 9, 11, and 12, Table 1).

**Scheme 1:**
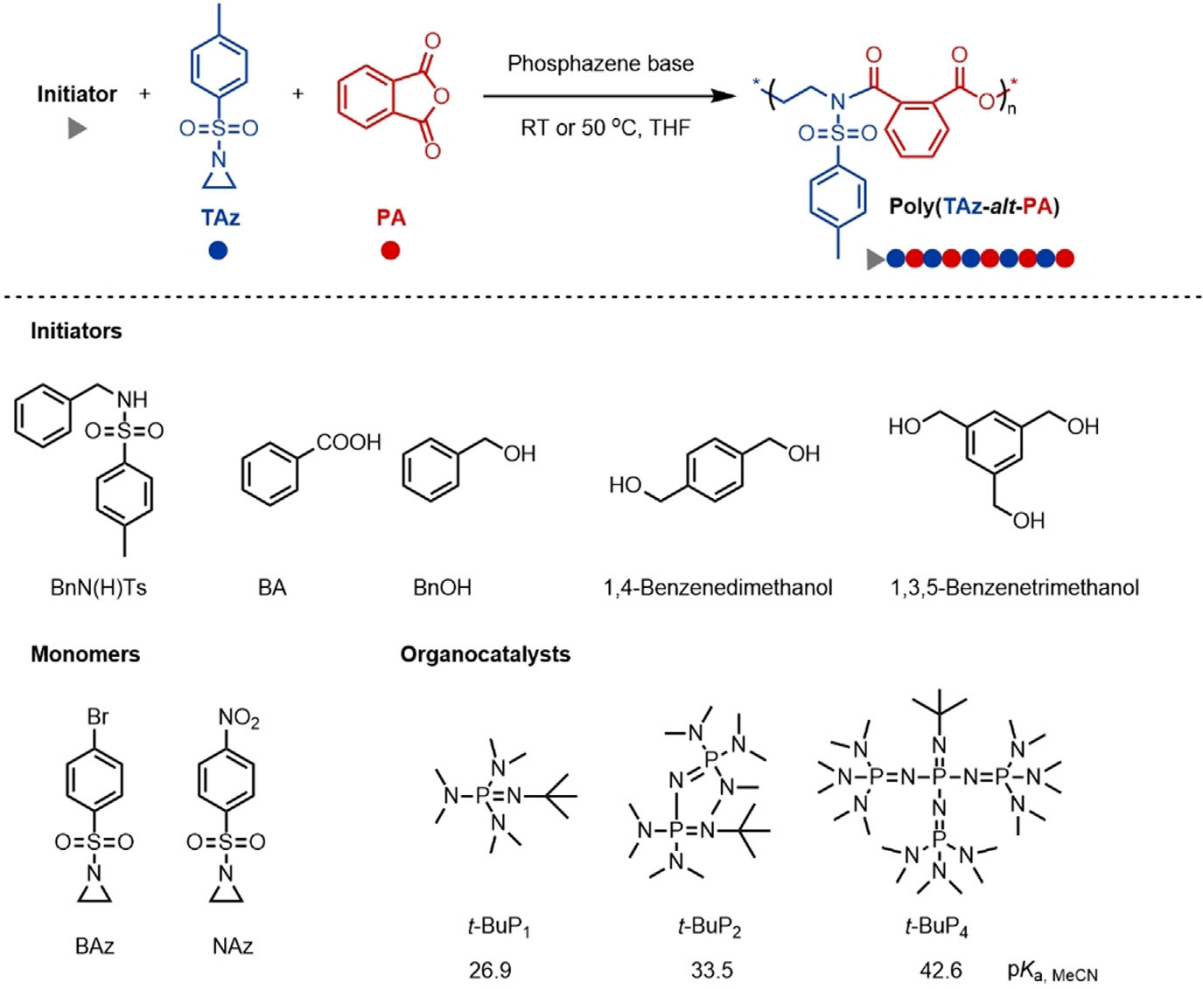
Copolymerizations of *N*‐sulfonyl aziridines with PA.^[a]^

FTIR characterization was used to confirm the structure of copolymers (Figure 1 b). The absorption peaks at 1721 cm^−1^ and 1691 cm^−1^ correspond to the stretching vibration of C=O in esters and C=O in amide units, respectively. The FTIR spectrum exhibits peaks at 1273 cm^−1^ and 1166 cm^−1^, corresponding to C‐N and C‐O stretching vibrations. The results confirm the presence of the ester and amide linkages in the copolymers. The chemical structures of the copolymers were further analyzed by ^1^H NMR and ^13^C NMR spectroscopy (Figure 1 c and Figure S1). The characteristic signal of the methylene protons of the TAz moves to a low field from 2.37 ppm to 4.45 ppm and 4.21 ppm after the formation of esters and amides linkages (e and f in Figure 1 c). Peaks corresponding to the methylene of BnN(H)Ts are observed at 5.03 ppm (a in Figure 1 c), indicating BnN(H)Ts as an effective initiator. We did not observe any amine linkage signals at 3.15–3.34 ppm, showing its perfectly alternating structure.

To further confirm the structure and terminal functional groups of poly(TAz‐*alt*‐PA), matrix‐assisted laser desorption ionization time‐of‐flight mass spectrometry (MALDI‐TOF MS) was used (Figure 1 d). Two main series of peaks are observed in accordance with sodium‐cationized poly(TAz‐*alt*‐PA), which contains BnN(H)Ts at one chain‐end, and TAz or PA at the other chain‐end. The molecular mass of adjacent peaks showed a fixed interval of 345.07, which is the molecular mass of the repeating unit of TAz and PA. The results show that the copolymers have a perfectly alternating nature having at one chain‐end BnN(H)Ts and at the other chain‐end either TAz or PA. No molecular mass corresponding to chains from inter‐ and intramolecular nucleophilic attacks was observed, indicating the absence of competitive side reactions. At high conversion when the concentration of the monomers is low, a slightly decreasing alternating degree was observed in MALDI‐TOF MS, but ^1^H NMR spectrum was not observed amine linkage signals and SEC traces remained narrow.

The less basic phosphazene bases, *t*‐BuP_1_ and *t*‐BuP_2_, caused lower reaction rates (Entry 5–8, Table 1). The alternating degree was not affected, but a slightly broader *Ð* was found by SEC (Figure S10–S13). The results show that the basicity of phosphazens mainly affects the rates and not molecular weight or *Ð*. Increasing the reaction temperature to 50 °C significantly accelerates ROAP (Entries 9 and 10, Table 1). Narrow and unimodal traces of SEC (Figure S14,S15) remain, even with prolonged reaction time. The ROAP of TAz with PA is fast, highly‐controlled, and highly‐selective at 50 °C without competitive side reactions. For further evaluation of the catalytic activity, the monomer/initiator feed ratios were increased to 50 and 100 (Entry 11 and 12, Table 1). Well‐controlled molecular weights with narrow distributions (*Ð*=1.04 and 1.02) were observed, indicating a highly controlled polymerization behavior.

Given the homopolymerizability of TAz, excess TAz as the “third” monomer could perform a one‐pot synthesis of P(TAz‐*alt*‐PA)‐*b*‐PTAz type diblock copolymers since homopolymerization of TAz will happen after complete consumption of PA. To closely monitor the copolymerization progress, we used a low‐reactive *t*‐BuP_2_ as the organocatalyst (Entry 13, Table 1). Excess TAz accelerated the progress of ROAP; complete conversion of PA was reached in 3 h. As demonstrated by in situ FTIR (Figure 2 a), the consistency of the decrease of PA and increase of esters and amides groups indicates an alternating copolymerization. As monitored by ^1^H NMR spectroscopy (Figure S9), PTAz peaks appeared and increased only after the complete consumption of PA. The molecular weight was increased while maintaining unimodal and narrow distributions (Figure S16), indicating the formation of a block sequence rather than mixtures of P(TAz‐*alt*‐PA) and PTAz.

Kinetic studies were performed to monitor the copolymerization progress (Figure 2). The conversion of PA shows a linear relationship with time (Figure 2 b), indicating a zero‐order dependence with respect to the concentration of PA. As shown in Figure 2 d–f, the kinetics reveal that the polymerization rate has a first‐order dependence of TAz concentration, a zero‐order dependence of the concentration of PA, and a first order dependence of *t*‐BuP_2_ concentration. Together, the data support the following rate law d[PA]/d*t*=*k*_obs_=*k*_p_[TAz][*t*‐BuP_2_]. The results indicate that the ring‐opening of TAz is the rate determined step. The evolution of molecular weight against TAz conversion showed a linear correlation with narrow distribution (*Ð*<1.10, Figure 2 c), suggesting a living behavior.

**Figure 2:**
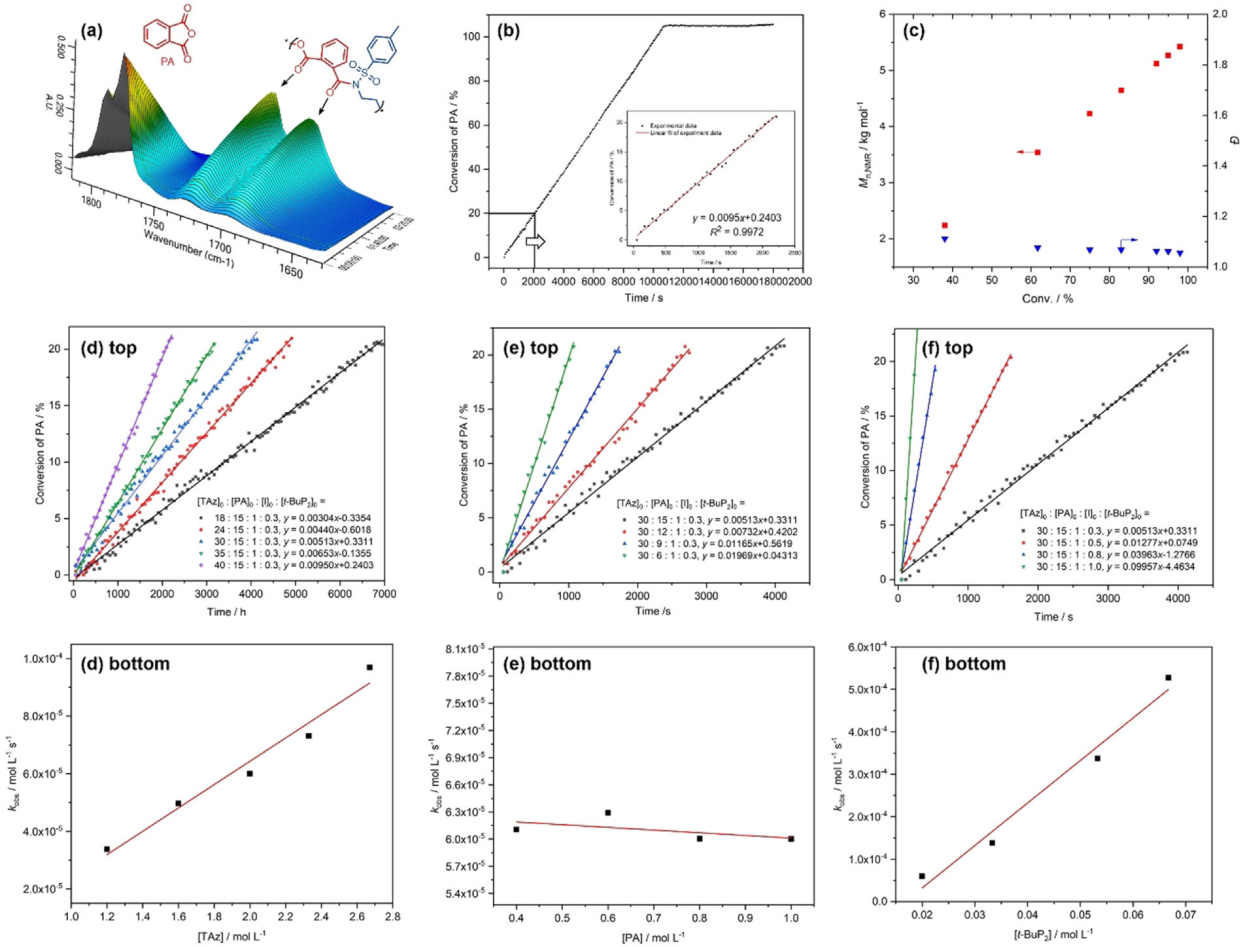
a,b) In‐situ FTIR surface image and the conversion of PA overtime for ROAP at a ratio of [TAz]_0_/[PA]_0_/[BnN(H)Ts]_0_/[*t*‐BuP_2_]=40/15/1/0.3. c) Molecular weight (*M*_n,NMR_) and molecular weight distribution (*Ð*) over TAz conversion for the ROAP; d–f) Kinetics of the ROAP of TAz with PA in situ FTIR at fixed time intervals of 1 min.

Based on the above studies, a plausible mechanism of the copolymerization of TAz with PA is proposed (Scheme 2). The super Brønsted base *t*‐BuP_4_ (p*K*_a_=42.6 in MeCN)^[13]^ abstracts a proton from the initiator, forming an active nitrogen anion, as supported by NMR titration experiments (Figure S30). The nitrogen anion attacks and ring‐opens the TAz, and the active anion center is transformed into amine chain end. Then, the amine anion easily attacks the higher reactive PA. The propagation begins through the anionic chain‐end as activating species to copolymerize the monomers (Scheme 2, Cycle 1). The self‐propagation of TAz occurs after full PA consumption (Scheme 2, Cycle 2).

**Scheme 2:**
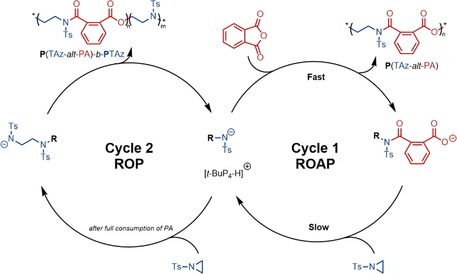
Plausible copolymerization pathways of TAz with PA towards poly(ester amide)‐based homo‐ and block copolymers.

The influence of the initiator structure was investigated (Entry 14–17, Table 1). Benzoic acid and benzyl alcohol showed practically the same catalytic efficiency, indicating that the carboxyl group and primary hydroxyl groups are the actual initiators. High polar DMF causes a faster reaction rate without breaking the alternating degree, and the low polar CH_2_Cl_2_ affords a lower reaction rate along with a slight decrease in the alternating degrees (Entry 18,19, Table 1). Other *N*‐sulfonyl aziridines were synthesized and examined for the ROAP with PA. A higher active *N*‐brosylaziridine (BAz) was copolymerized resulting in a material with narrow molecular weight distribution (Entry 20, Table 1, Figure S23). However, the higher activity of *N*‐(4‐nitrobenzenesulfonyl)aziridine (NAz) from PA, produced an insoluble white powder, due to the presence of insoluble blocks of repeating units of NAz,^[15]^ indicating lower alternating degrees.

The resulted poly(ester amide)s were analyzed by thermogravimetric analysis and differential scanning calorimetry (Figure 3), showing a *T*_d 5 %_ of 265 °C and a *T*_g_ of 114 °C. The thermal stability is almost the same as that of the poly(propylene oxide‐*alt*‐PA) (*T*_d 5 %_ of 269 °C),^[16]^ while the *T*_g_ of poly(TAz‐*alt*‐PA) significantly increase than the poly(propylene oxide‐*alt*‐PA) (*T*_g_ of 55 °C),^[17]^ because of amide linkages. Compared with poly[(3S)‐3‐benzyl 6‐methyl‐morpholine‐2, 5‐dione] from ROP of (3S)‐3‐benzyl 6‐methyl‐morpholine‐2, 5‐dione (*T*_d 5 %_ of 295 °C, *T*_g_ of 100 °C),^[4a]^ the decreasing in thermal stability was observed, and the *T*_g_ of poly(TAz‐*alt*‐PA) increases under the influence of the benzene ring in backbone.

**Figure 3:**
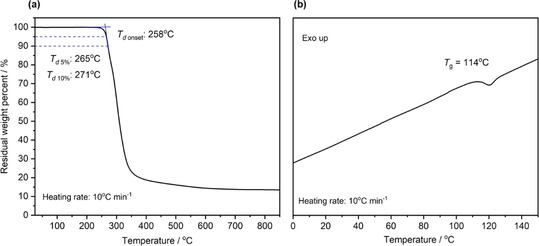
a) TGA thermogram for poly(TAz‐*alt*‐PA) under N_2_; b) DSC thermogram for poly(TAz‐*alt*‐PA) under N_2_.

In conclusion, we report a new synthetic methodology of poly(ester amide)s from ROAP of *N*‐sulfonyl aziridines with cyclic anhydrides. The commercially available phosphazene organocatalysts were used to perform a highly‐active, highly‐controlled, and highly‐selective copolymerization without any competitive side reactions (zwitterionic mechanism and exchange transacylations). Poly(ester amide)s are obtained in an energy‐saving way (temperature ≤50 °C) with perfectly alternating sequence distribution, highly controlled molecular weight, and narrow molecular weight distribution (*Ð*<1.10). The copolymerization progress has a first‐order dependence with *N*‐sulfonyl aziridines concentration, a zero‐order dependence with cyclic anhydrides concentration, and a first‐order dependence with phosphazenes concentration. The effects of temperature, solvent, initiator, and basicity of phosphazenes were studied. Two catalytic cycles involving ROAP of *N*‐sulfonyl aziridines with cyclic anhydrides and ROP of *N*‐sulfonyl aziridines were proposed to explain the synthesis of alternating copolymers and diblock copolymers in one‐pot.

## Conflict of interest

The authors declare no conflict of interest.

## Supporting information

As a service to our authors and readers, this journal provides supporting information supplied by the authors. Such materials are peer reviewed and may be re‐organized for online delivery, but are not copy‐edited or typeset. Technical support issues arising from supporting information (other than missing files) should be addressed to the authors.

Supplementary
