# Supplementary material for: Well‐Defined Poly(Ester Amide)‐Based Homo‐ and Block Copolymers by One‐Pot Organocatalytic Anionic Ring‐Opening Copolymerization of N‐Sulfonyl Aziridines and Cyclic Anhydrides
Source: Angew Chem Int Ed Engl. 2021 Feb 24;60(13):6949–54. doi: 10.1002/anie.202015339 (PMC8048504; doi:10.1002/anie.202015339)
Supplement: Supplementary file 1 — Supplementary [file ANIE-60-6949-s001.pdf]

Angewandte Chemie
Eine Zeitschrift der Gesellschaft Deutscher Chemiker

Supporting Information

Well-Defined Poly(Ester Amide)-Based Homo- and Block Copolymers by One-Pot Organocatalytic Anionic Ring-Opening Copolymerization of *N*-Sulfonyl Aziridines and Cyclic Anhydrides

*Jiaxi Xu and Nikos Hadjichristidis**

anie_202015339_sm_miscellaneous_information.pdf

Contents

1. Materials 4

2. Instrumentation 4

3. Methods 5

4. Introduction of competitive side reactions 8

Scheme S1. Competitive side reactions including: (a) zwitterionic mechanisms; (b) intermolecular and intramolecular exchange transacylations. 8

5. NMR spectra for copolymers 8

Figure S1. ^{13}C NMR (100 MHz, $CDCl_3$, 25°C) spectrum of poly(TAz-*alt*-PA). 8

Figure S2. ^{1}H NMR (400 MHz, $CDCl_3$, 25°C) spectrum of PTAz in $CDCl_3$. 9

Figure S3. ^{1}H NMR (400 MHz, $CDCl_3$, 25°C) spectrum of the copolymer of TAz and PA initiated by benzoic acid (Entry 14). 9

Figure S4. ^{1}H NMR (400 MHz, $CDCl_3$, 25°C) spectrum of the copolymers of TAz and PA initiated by benzyl alcohol (Entry 15) 10

Figure S5. ^{1}H NMR (400 MHz, $CDCl_3$, 25°C) spectrum of the copolymers of TAz and PA initiated by 1,4-benzenedimethanol (Entry 16). 10

Figure S6. ^{1}H NMR (400 MHz, $CDCl_3$, 25°C) spectrum of the copolymers of TAz and PA initiated by 1,3,5-benzenetrimethanol (Entry 17). 11

Figure S7. ^{1}H NMR (400 MHz, $CDCl_3$, 25 ^{0}C) spectrum of the copolymers of BAz and PA (Entry 20). 11

Figure S8. ^{1}H NMR (400 MHz, $CDCl_3$, 25°C) spectrum of P(PTAz-*alt*-PA)-*b*-PTAz block copolymers 12

Figure S9. ^{1}H NMR (400 MHz, $CDCl_3$, 25°C) spectra at different reaction times from the copolymerization of TAz and PA performed in THF ($[PA]_0$ = 1 M) at 25°C at a ratio of $[TAz]_0/[PA]_0/[BnN(H)Ts]_0/[t\text{-}BuP_2]$ = 40/15/1/0.3 which leads to a P(PTAz-*alt*-PA)-*b*-PTAz block copolymer (Entry 14). 12

6. SEC traces for copolymers 13

Figure S10. SEC trace for the copolymers of TAz and PA ($[TAz]_0/[PA]_0/[BnN(H)Ts]_0/[t\text{-}BuP_2]_0$ = 15/15/1/0.3, 24 h) (Entry 5). 13

Figure S11. SEC trace of the copolymers of TAz and PA ($[TAz]_0/[PA]_0/[BnN(H)Ts]_0/[t\text{-}BuP_2]_0$ = 15/15/1/0.3, 48 h) (Entry 6). 13

Figure S12. SEC trace of the copolymers of TAz and PA ($[TAz]_0/[PA]_0/[BnN(H)Ts]_0/[t\text{-}BuP_1]_0$ = 15/15/1/0.3, 24 h) (Entry 7). 14

Figure S13. SEC trace of the copolymers of TAz and PA ($[TAz]_0/[PA]_0/[BnN(H)Ts]_0/[t\text{-}BuP_1]_0$ = 15/15/1/0.3, 48 h) (Entry 8). 14

Figure S14. SEC trace of the copolymers of TAz and PA ($[TAz]_0/[PA]_0/[BnN(H)Ts]_0/[t\text{-}BuP_4]_0$ = 15/15/1/0.3, 50°C, 1 h) (Entry 9). 15

Figure S15. SEC trace of the copolymers of TAz and PA ($[TAz]_0/[PA]_0/[BnN(H)Ts]_0/[t\text{-}BuP_4]_0$ = 15/15/1/0.3, 50°C, 24 h) (Entry 10). 15

Figure S16. SEC traces of P(TAz-*alt*-PA) and P(PTAz-*alt*-PA)-*b*-PTAz diblock copolymers. (The black one in Entry 13) 16

Figure S17. SEC trace of the copolymers of TAz and PA ($[TAz]_0/[PA]_0/[BA]_0/[t\text{-}BuP_2]_0$ = 15/15/1/0.3, 25°C, 24 h) (Entry 14). 16

Figure S18. SEC trace of copolymers of TAz and PA ($[TAz]_0/[PA]_0/[BnOH]_0/[t\text{-}BuP_2]_0$ = 15/15/1/0.3, 25°C, 24 h) (Entry 15). 17

Figure S19. SEC trace of copolymers of TAz and PA ($[TAz]_0/[PA]_0/[\text{1,4-benzenedimethanol}]_0/[t\text{-}BuP_2]_0$ = 15/15/1/0.6, 25°C, 24 h) (Entry 16). 17

Figure S20. SEC trace of the copolymers of TAz and PA ($[TAz]_0/[PA]_0/[\text{1,3,5-benzenetrimethanol}]_0/[t\text{-}BuP_2]_0$ = 15/15/1/0.9, 25°C, 24 h) (Entry 17). 18

Figure S21. SEC trace of the copolymers of TAz and PA ($[TAz]_0/[PA]_0/[BnN(H)Ts]_0/[t\text{-}BuP_2]_0$ = 15/15/1/0.3, in DMF, 25°C, 20 h) (Entry 18). 18

Figure S22. SEC trace of the copolymers of TAz and PA ($[TAz]_0/[PA]_0/[BnN(H)Ts]_0/[t\text{-}BuP_2]_0$ = 15/15/1/0.3, in CH_2Cl_2, 25°C, 24 h) (Entry 19). 19

Figure S23. SEC trace of the copolymers of BAz and PA ($[BAz]_0/[PA]_0/[BnN(H)Ts]_0/[t\text{-}BuP_4]_0$ = 15/15/1/0.3, in THF, 25°C, 12 h) (Entry 20). 19

7. FTIR for copolymers 20

Figure S24. FTIR spectrum of the copolymer of BAz and PA. 20

Figure S25. FTIR spectrum of the copolymer of NAz and PA. 20

Figure S26. FTIR spectrum of PTAz. 21

8. Kinetic study 21

Table S1. Experimental data for kinetic copolymerization experiments. 21

Figure S27. These reactions were performed by t-BuP$_2$ as a catalyst in THF ($[TAz]_0$ = 2 M) at 25°C with different amount of PA. The automatic sampling interval of *in-situ* FTIR is 1 min before 8 h, 5min after 8 h. 22

Figure S28. These reactions were performed by t-BuP$_2$ as a catalyst in THF ($[PA]_0$ = 1 M) at 25°C with different amount of TAz. The automatic sampling interval of *in-situ* FTIR is 1 min before 8 h, 5 min after 8 h. 22

Figure S29. These reactions were performed by t-BuP$_2$ as a catalyst in THF ($[PA]_0$ = 1 M) at 25°C with different amount of t-BuP$_2$. The automatic sampling interval of *in-situ* FTIR is 1 min before 8 h, 5 min after 8 h. 23

9. NMR titration for mechanism 23

Figure S30. ^{1}H NMR (400 MHz, $CDCl_3$, 25°C) spectra of (a) t-BuP$_4$, (b) BnN(H)Ts, and (c) t-BuP$_4$: BnN(H)Ts = 1 : 1 in $CDCl_3$ at same concentration of 0.08 mol L^{-1}. 23

10. NMR spectra of reactants 24

Figure S31. ^{1}H NMR (400 MHz, $CDCl_3$, 25°C) spectrum of TAz 24
Figure S32. ^{1}H NMR (400 MHz, $CDCl_3$, 25°C) spectrum of BnN(H)Ts 24
Figure S33. ^{1}H NMR (400 MHz, $CDCl_3$, 25°C) spectrum of BAz 25
Figure S34. ^{13}C NMR (100 MHz, $CDCl_3$, 25°C) spectrum of BAz 25
Figure S35. ^{1}H NMR (400 MHz, $CDCl_3$, 25°C) spectrum of NAz 26
Figure S36. ^{13}C NMR (100 MHz, $CDCl_3$, 25°C) spectrum of NAz 26

1. Materials

All operations were carried out inflamed Schlenk-type glassware in an inert atmosphere of argon or in an argon-filled glovebox. *tert*-Butylimino-tris-(dimethyl-amino)phosphorene (*t*-BuP$_1$), 1-*tert*-butyl-2,2,4,4,4-pentakis(dimethylamino)-2λ^5,4λ^5-catenadi(phosphazene) (*t*-BuP$_2$) (2.0 M in THF), and 1-*tert*-butyl-4,4,4-tris(dimethylamino)-2,2-bis[tris(dimethylamino)phosphoranylid-enamino]-2λ^5,4λ^5-catenadi(phosphazene) (*t*-BuP$_4$) (0.8 M in *n*-hexane) were supplied by Aldrich Chemicals and used without further purification. Benzyl alcohol (BnOH, anhydrous, 99.8%) was dried over calcium hydride (CaH_2) overnight, distilled, and then stored in a glovebox for use. Benzoic acid, *N*-tosylaziridine (TAz, 98 %), 1,4-benzenedimethanol, and 1,3,5-benzenetrimethanol were dried over phosphorus pentoxide. *N*-brosylaziridine (BAz) and *N*-(4-nitrobenzenesulfonyl)aziridine (NAz) were synthesized according to the literature.[1] Phthalic anhydride (PA, Aldrich ⩾ 99%) was purified by heating a 10 wt.% solution of PA in $CHCl_3$ to reflux for 30 min, followed by hot filtration through Celite. The filtration of PA was recrystallized at room temperature. Then the PA was sublimated at 100 °C twice under dynamic vacuum. Then the PA was freezing-drying by 1, 4-dioxane, and the trace amount of water was removed under vacuum in the presence of P_2O_5 for two days. Tetrahydrofuran (THF) was dried over sodium metal/benzophenone mixture and distilled, then stored in a glovebox for use. Dichloromethane (DCM) and dimethylformamide (DMF) were dried over CaH_2 overnight and distilled and then stored in a glovebox for use. Other chemicals were purchased from Aldrich Chemicals and used as received unless stated otherwise.

2. Instrumentation

Nuclear magnetic resonance (^{1}H NMR and ^{13}C NMR) measurements were recorded on a Bruker AVANCE III-400 MHz instruments. Size exclusion chromatography (SEC) measurements were designed at 35°C with THF as an eluent at a flow rate of 1.0 mL min^{-1}, installed with a Viscotek GPC$_{max}$ VE2001 system, and PSS columns (Styragel HR 3, 4 and 5). The dispersity (M_w/M_n, $Đ$) was tested by conventional SEC analysis under a calibration curve from polystyrene standards. Matrix-assisted laser desorption ionization time-of-flight (MALDI-ToF) mass measurements were recorded in linear mode, using 2,5-dihydroxybenzoic acid (DHB) as the matrix in THF

(sample/matrix: 1/25) and sodium iodide as an ionizing agent. *In-situ* Fourier-transform infrared spectroscopy (FTIR) study was conducted using a ReactIR 45m (Mettler Toledo). The samples were collected every 1 min before 8 h and 5 min after 8 h. Each spectrum was scanned 256 times. Thermogravimetric analysis (TGA) experiments were performed on a TGA Q500 analyzer (TA Instruments). Samples were heated from 25°C to 850°C (a heating rate of 10°C min^{-1}) under a nitrogen atmosphere. Differential scanning calorimetry (DSC) measurements were performed at a heating rate of 10°C min^{-1} on a Mettler Toledo DSC1/TC100 system under a nitrogen atmosphere. The curve of the second heating scan was adopted to determine the glass transition temperature (T_g).

3. Methods

Synthesis of *N*-brosylaziridine (BAz)[1c]

Ethanolamine (1.52 g, 25 mmol, 1.0 equiv) in 40 mL pyridine was added in a flamed-dried round-bottomed flask. After the mixture was cooled to -40°C, 4-bromobenzenesulfonyl chloride (13.29 g, 52 mmol, 2.2 equiv) in 20 mL pyridine was added dropwise into the mixture at -40°C with stirring for 30 min. The reaction mixture was stirred at room temperature for 5 h. A mixture of water/ice was added to the mixture. The residue was filtered off and kept aside. The filtrate was extracted with CH_2Cl_2 (2 x 40 mL). The combined organic layer was dried over Na_2SO_4, filtered, and concentrated under vacuum. The crude product [2-((4-bromophenyl)sulfonamido)ethyl 4-bromobenzenesulfonate] was purified on a silica gel column chromatography (R_f = 0.24, EtOAc/petroleum ether = 1/3) as a white solid (8.75 g, 70 %).

The previous product [2-((4-bromophenyl)sulfonamido)ethyl 4-bromobenzenesulfonate] (8.75 g, 17.5 mmol, 1.0 equiv) was dissolved in toluene (100 mL). A 20 wt. % solution of KOH (3.34 g in 25 mL, 60 mmol, 3.4 equiv) was added slowly. After 1 h, the organic phase was separated and washed with water until a neutral pH of the aqueous phase. The organic phase was dried over anhydrous Na_2SO_4, filtered, and concentrated under vacuum. The crude product was purified by column chromatography (R_f = 0.4, EtOAc/petroleum ether = 1/3) to afford the desired compound as a white solid (4.36 g, 95%). ^{1}H NMR ($CDCl_3$): 7.82 (d, *J* = 8.6 Hz, 2H), 7.70 (d, *J* = 8.6 Hz, 2H), 2.41 (s, 4H). ^{13}C NMR ($CDCl_3$): 137.2, 132.6, 129.6, 129.0, 27.2.

Synthesis of *N*-(4-nitrobenzenesulfonyl)aziridine (NAz)[1a, 1b]

Ethanolamine (2.68 g, 44 mmol, 1.0 equid) and Et_3N (2.0 mL, 13.84 mmol, 0.32 equid) in 40 mL anhydrous CH_2Cl_2 were added in a flamed-dried round-bottomed flask. After the mixture was cooled to 0°C, 4-nitrobenzenesulfonylchloride (9.76 g, 44 mmol, 1.0 equid) in anhydrous CH_2Cl_2 (10 mL) were added into the mixture. The reaction mixture was stirred at room temperature for 18 h. The solvent was removed under reduced pressure before adding EtOAc (130 mL). The organic layer was washed by 1M aqueous NaOH (40 mL), saturated aqueous $NaHCO_3$ (40 mL), brine (40 mL), and dried over anhydrous Na_2SO_4, filtered and concentrated. The crude product *N*-(2-hydroxyethyl)-4-nitrobenzenesulfonamide was purified on a silica gel column chromatography (R_f = 0.22, EtOAc/petroleum ether = 1/1) as pale yellow solid (8.51 g, 79%). ^{1}H NMR (DMSO-d_6): 8.41 (d, *J* = 8.8 Hz, 2H), 8.05 (d, *J* = 8.8 Hz, 2H), 4.73 (t, *J* = 5.5 Hz, 1H), 3.35 (m, 2H), 2.86 (q, *J* = 6.1 Hz, 2H).

The previous product *N*-(2-hydroxyethyl)-4-nitrobenzenesulfonamide (8.51 g, 34.56 mmol, 1.0 equid) and Et_3N (6.02 mL, 41.47 mmol, 1.2 equid) in 85 mL anhydrous CH_2Cl_2 were added in a flamed-dried round-bottomed flask. After 5 min of stirring, ethylsulfonyl chloride (4.43 g, 34.56 mmol, 1.0 equid) in 10 mL anhydrous CH_2Cl_2 was added dropwise into the mixture at 0°C with stirring for 30 min. The reaction mixture was stirred at room temperature for 18 h. The solvent was removed under reduced pressure before adding EtOAc (420 mL). The organic layer was washed by saturated aqueous $NaHCO_3$ (90 mL), brine (90 mL), and dried over anhydrous Na_2SO_4, filtered, and concentrated. The crude product 2-((4-nitrophenyl)sulfonamido)ethyl ethanesulfonate was purified on a silica gel column chromatography (R_f = 0.23, EtOAc/petroleum ether = 2/3) as pale yellow solid (4.18 g, 36%). ^{1}H NMR ($CDCl_3$): 8.38 (d, *J* = 8.8 Hz, 2H), 8.07 (d, *J* = 8.8 Hz, 2H), 5.22 (t, *J* = 6.0 Hz, 1H), 4.29 (t, *J* = 5.1 Hz. 2H), 3.43-3.39 (m, 2H), 3.16 (q, *J* = 7.4 Hz, 2H), 1.42 (t, *J* = 7.5 Hz, 3H).

The previous product 2-((4-nitrophenyl)sulfonamido)ethyl ethanesulfonate (4.18 g, 12.35 mmol, 1.0 equid) was dissolved in 85 mL benzene. A solution of KOH (4.09 g in 22 mL H_2O, 7.14 mmol, 0.58 equiv) was added slowly. After 1 h, the solvent was removed under reduced pressure before adding EtOAc (250 mL). The organic phase was washed with water (60 mL), brine (60 mL), and

dried over anhydrous Na_2SO_4, filtered, and concentrated under vacuum. The crude product was purified by column chromatography (R_f = 0.27, EtOAc/petroleum ether = 1/3,) to afford the desired compound as a pale yellow solid (2.54 g, 90%). ^{1}H NMR ($CDCl_3$): 8.41 (d, *J* = 8.8 Hz, 2H), 8.17 (d, *J* = 8.8 Hz, 2H), 2.49 (s, 4H).

General procedure for the copolymerization of *N*-sulfonyl aziridines and PA

In an argon-filled glovebox, PA (0.088 g, 0.6 mmol), TAz (0.116 g, 0.6 mmol), and initiator (*N*-benzyl-4-methylbenzenesulfonamide) (0.01045 g, 0.04 mmol) were dissolved in 0.6 mL anhydrous THF. After 5 min of stirring, phosphazene base was added to start the polymerization. The mixture was stirred at 25°C or 50°C under the argon atmosphere. The entire polymerization was monitored by ^{1}H NMR and *in-situ* FTIR. The product polymer was precipitated by the slow addition of the reaction solution to an excess of methanol. The white precipitate was collected via centrifugation, purified by reprecipitation in methanol, and dried at 25°C in a vacuum oven. The complete consumption of monomer was confirmed by ^{1}H NMR in these cases.

4. Introduction of competitive side reactions

Competitive side reactions:

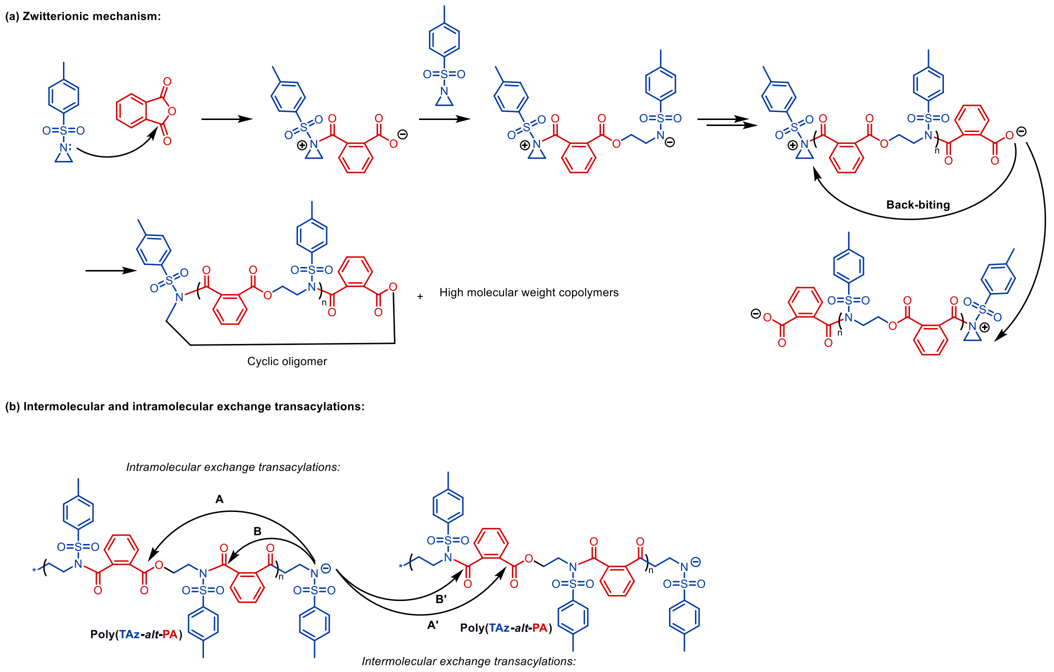

Scheme S1. Competitive side reactions including: (a) zwitterionic mechanisms; (b) intermolecular and intramolecular exchange transacylations.

5. NMR spectra for copolymers

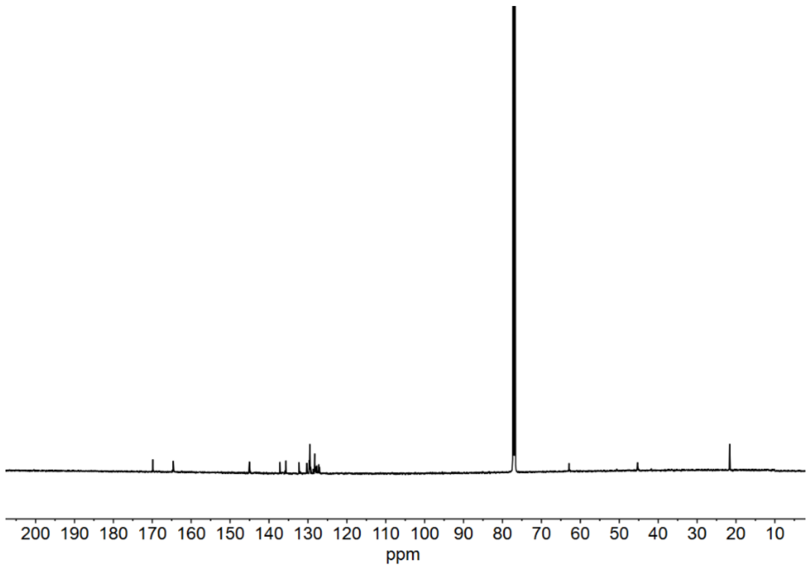

Figure S1. ^{13}C NMR (100 MHz, $CDCl_3$, 25°C) spectrum of poly(TAz-*alt*-PA).

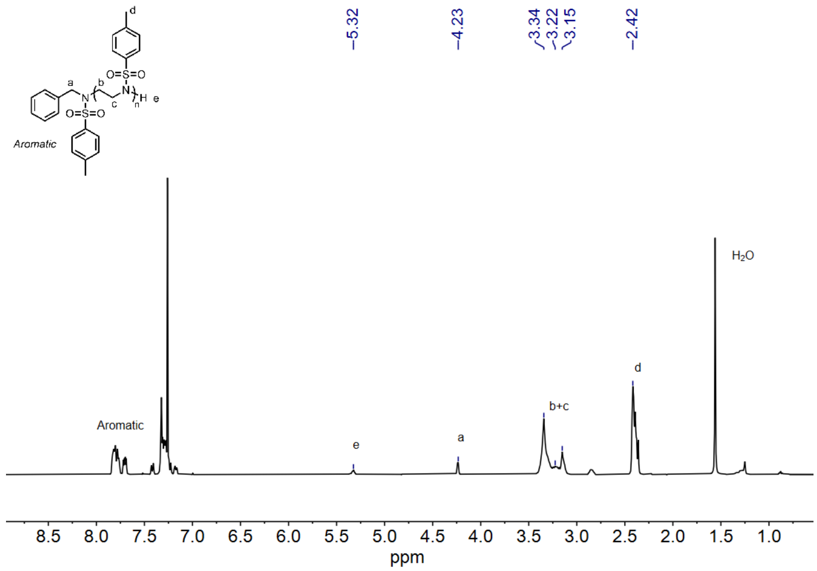

Figure S2. ^{1}H NMR (400 MHz, $CDCl_3$, 25°C) spectrum of PTAz in $CDCl_3$.

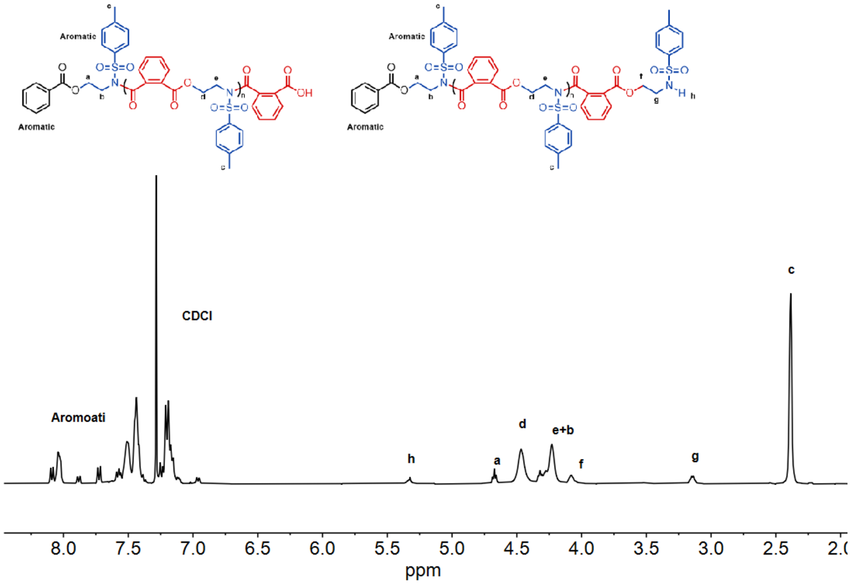

Figure S3. ^{1}H NMR (400 MHz, $CDCl_3$, 25°C) spectrum of the copolymer of TAz and PA initiated by benzoic acid (Entry 14).

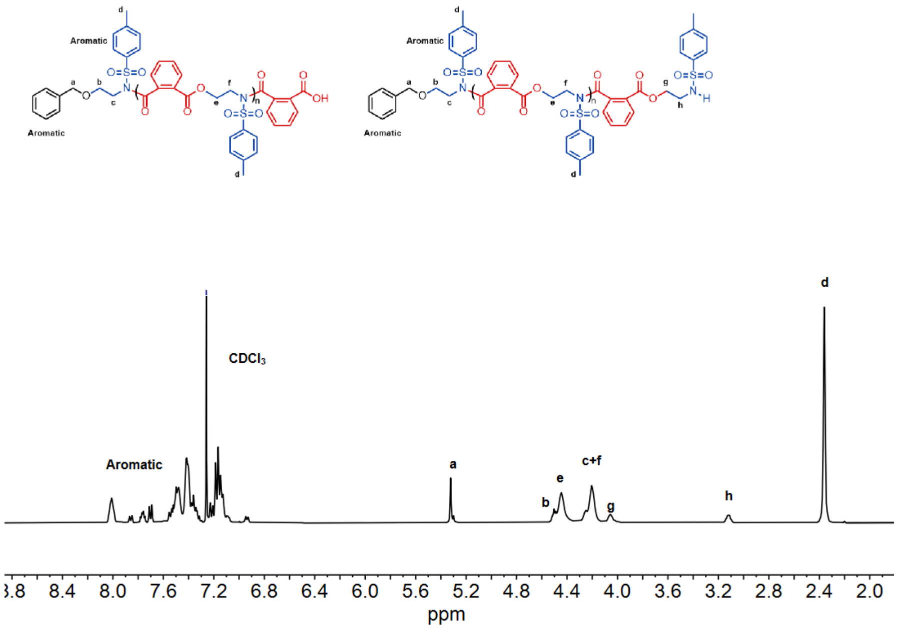

Figure S4. ¹H NMR (400 MHz, $CDCl_3$, 25°C) spectrum of the copolymers of TAz and PA initiated by benzyl alcohol (Entry 15).

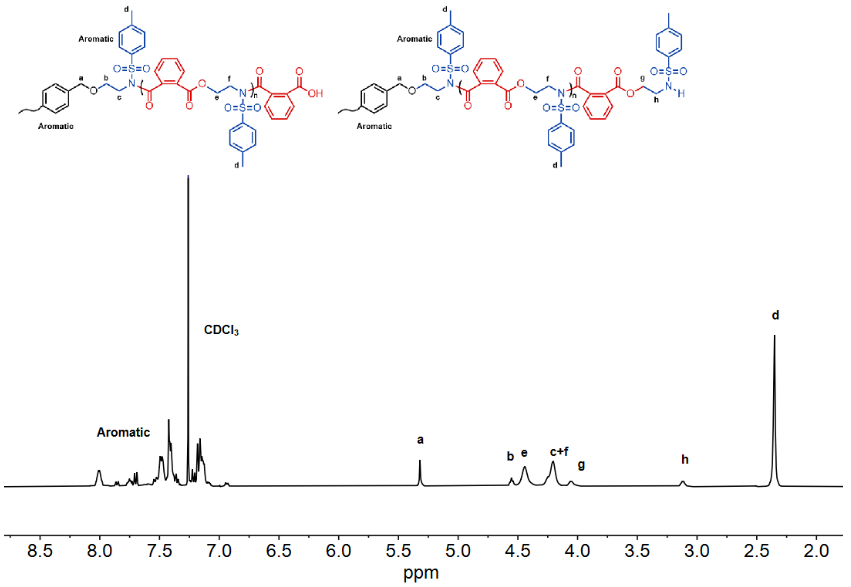

Figure S5. ¹H NMR (400 MHz, $CDCl_3$, 25°C) spectrum of the copolymers of TAz and PA initiated by 1,4-benzenedimethanol (Entry 16).

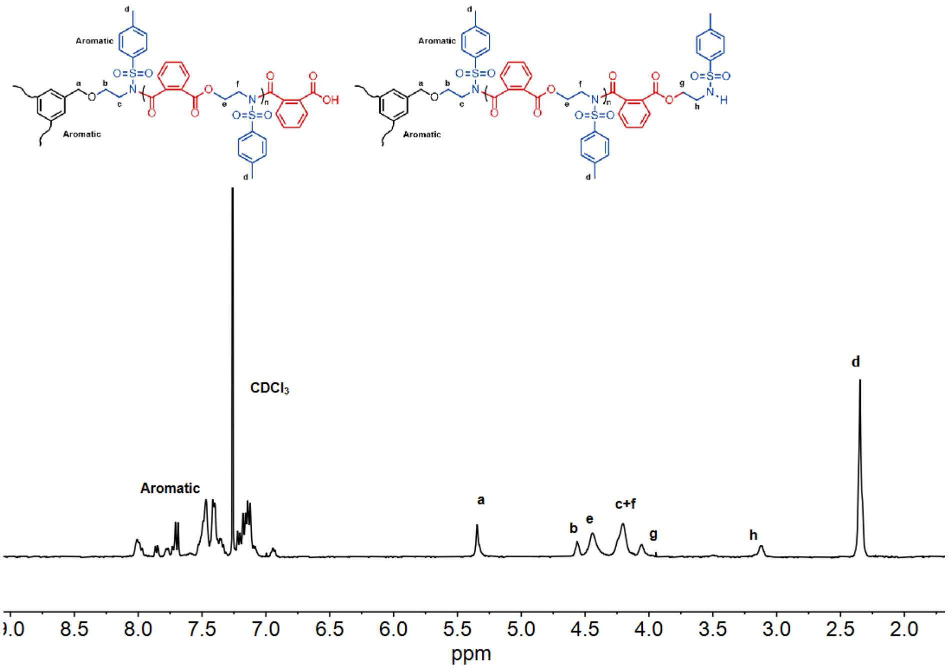

Figure S6. ^{1}H NMR (400 MHz, $CDCl_3$, 25°C) spectrum of the copolymers of TAz and PA initiated by 1,3,5-benzenetrimethanol (Entry 17).

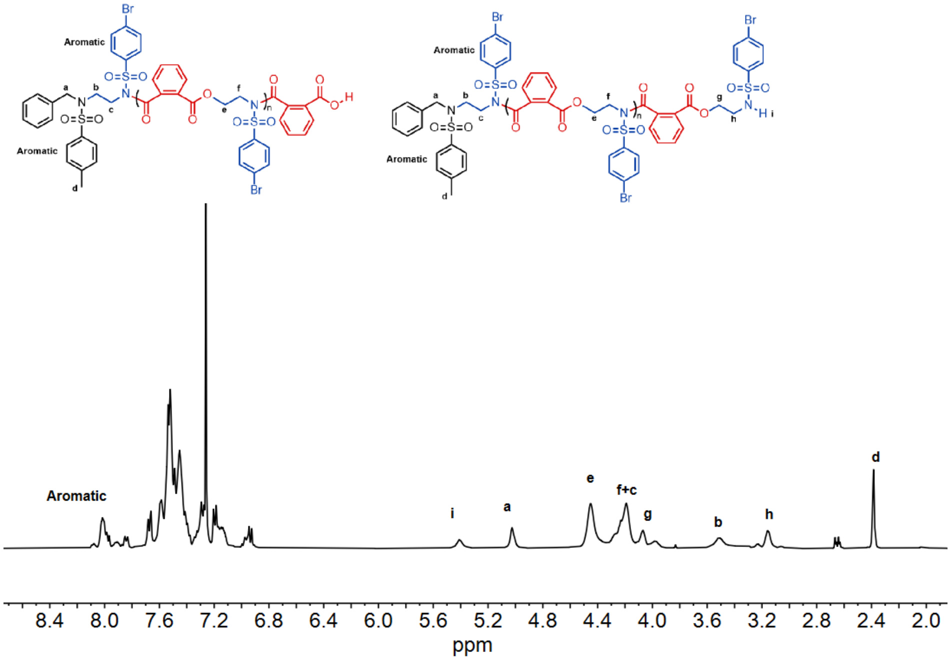

Figure S7. ^{1}H NMR (400 MHz, $CDCl_3$, 25 ^{0}C) spectrum of the copolymers of BAz and PA (Entry 20).

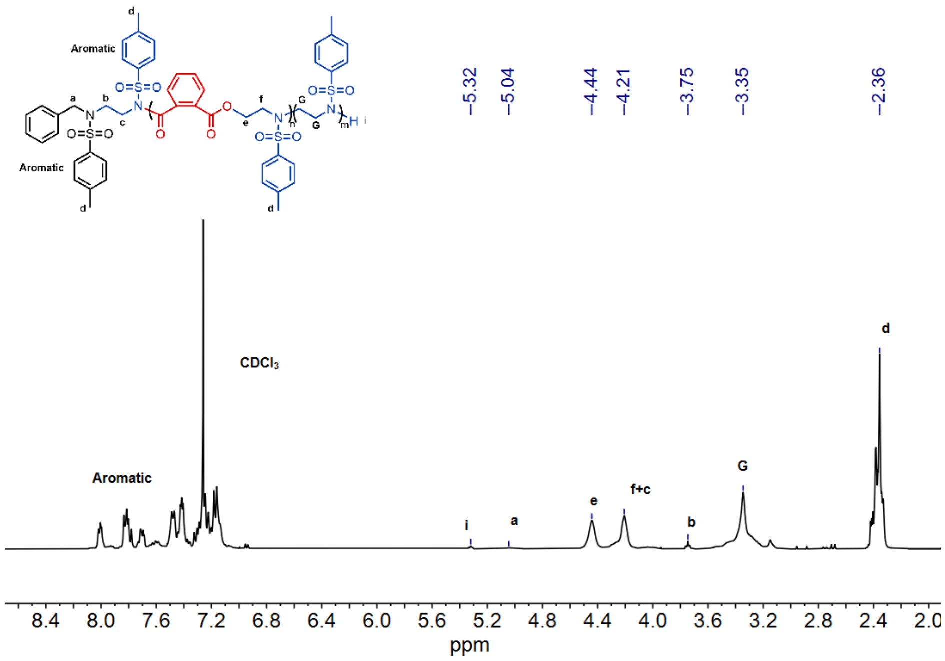

Figure S8. ^{1}H NMR (400 MHz, $CDCl_3$, 25°C) spectrum of P(PTAz-*alt*-PA)-*b*-PTAz block copolymers.

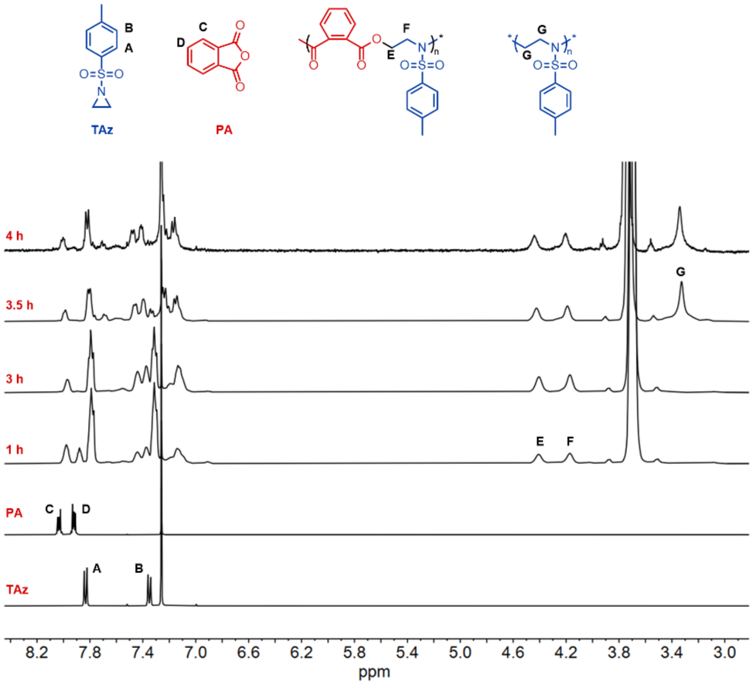

Figure S9. ^{1}H NMR (400 MHz, $CDCl_3$, 25°C) spectra at different reaction times from the copolymerization of TAz and PA performed in THF ($[PA]_0$ = 1 M) at 25°C at a ratio of $[TAz]_0/[PA]_0/[BnN(H)Ts]_0/[t\text{-}BuP_2]$ = 40/15/1/0.3 which leads to a P(PTAz-*alt*-PA)-*b*-PTAz block copolymer (Entry 14).

6. SEC traces for copolymers

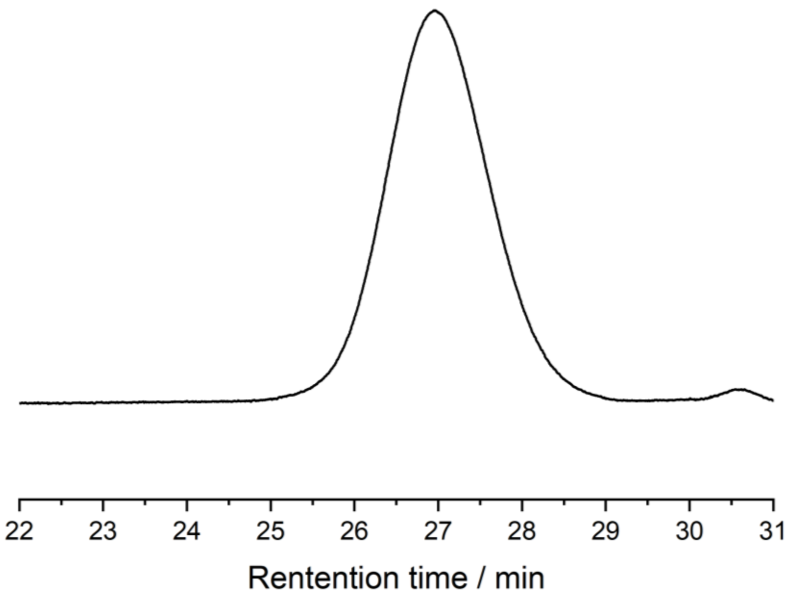

Figure S10. SEC trace for the copolymers of TAz and PA ($[TAz]_0/[PA]_0/[BnN(H)Ts]_0/[t\text{-}BuP_2]_0$ = 15/15/1/0.3, 24 h) (Entry 5).

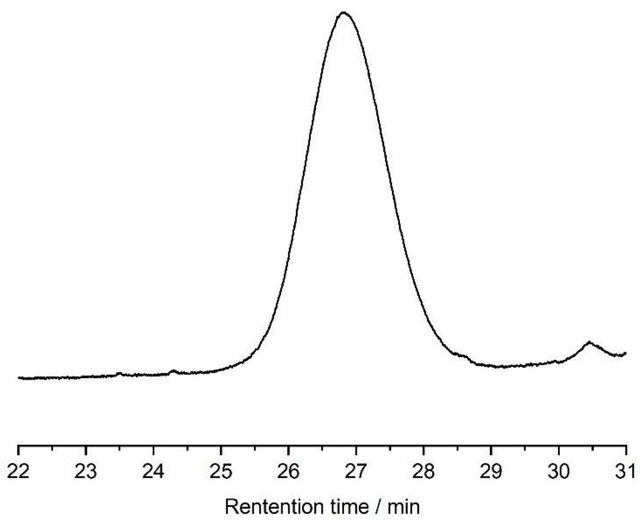

Figure S11. SEC trace of the copolymers of TAz and PA ($[TAz]_0/[PA]_0/[BnN(H)Ts]_0/[t\text{-}BuP_2]_0$ = 15/15/1/0.3, 48 h) (Entry 6).

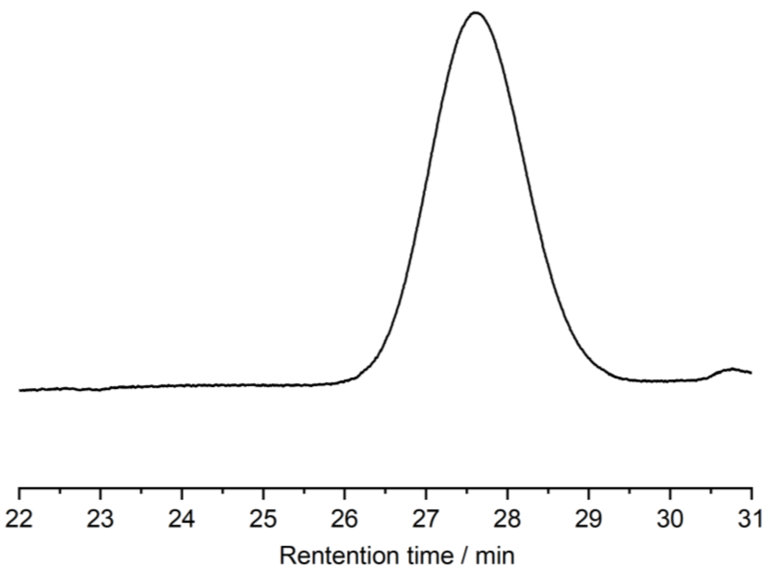

Figure S12. SEC trace of the copolymers of TAz and PA ($[TAz]_0/[PA]_0/[BnN(H)Ts]_0/[t\text{-}BuP_1]_0$ = 15/15/1/0.3, 24 h) (Entry 7).

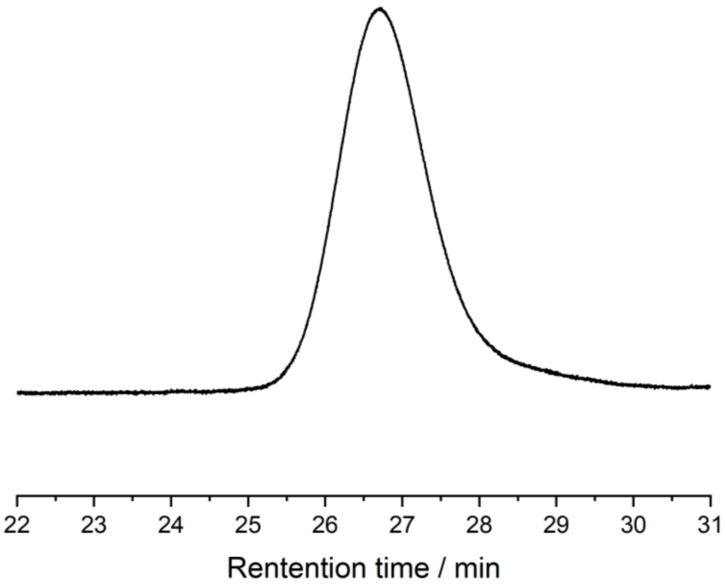

Figure S13. SEC trace of the copolymers of TAz and PA ($[TAz]_0/[PA]_0/[BnN(H)Ts]_0/[t\text{-}BuP_1]_0$ = 15/15/1/0.3, 48 h) (Entry 8).

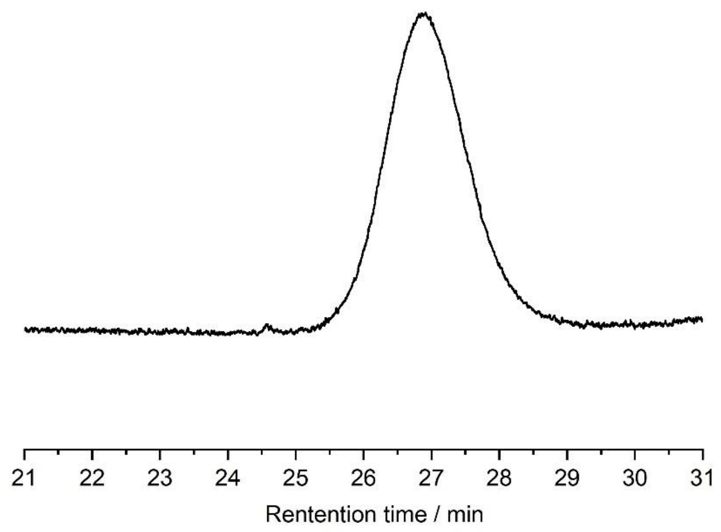

Figure S14. SEC trace of the copolymers of TAz and PA ($[TAz]_0/[PA]_0/[BnN(H)Ts]_0/[t\text{-}BuP_4]_0$ = 15/15/1/0.3, 50°C, 1 h) (Entry 9).

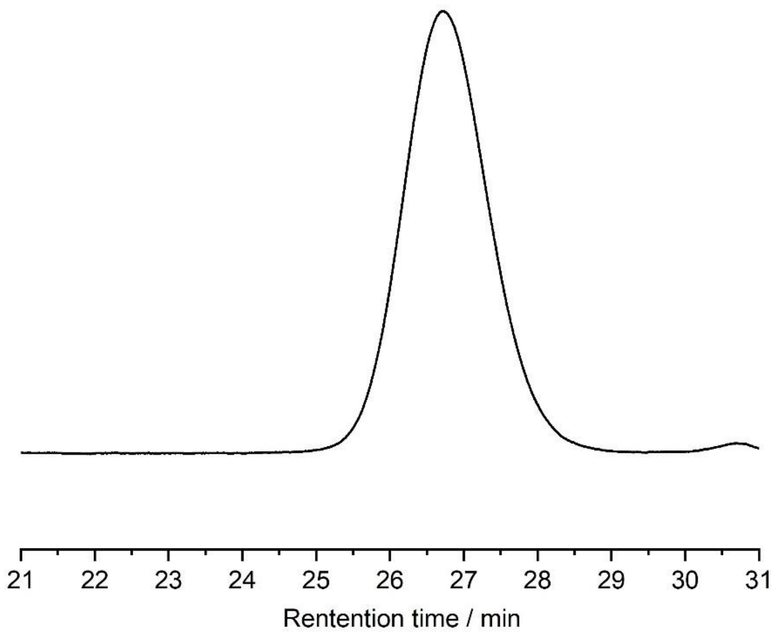

Figure S15. SEC trace of the copolymers of TAz and PA ($[TAz]_0/[PA]_0/[BnN(H)Ts]_0/[t\text{-}BuP_4]_0$ = 15/15/1/0.3, 50°C, 24 h) (Entry 10).

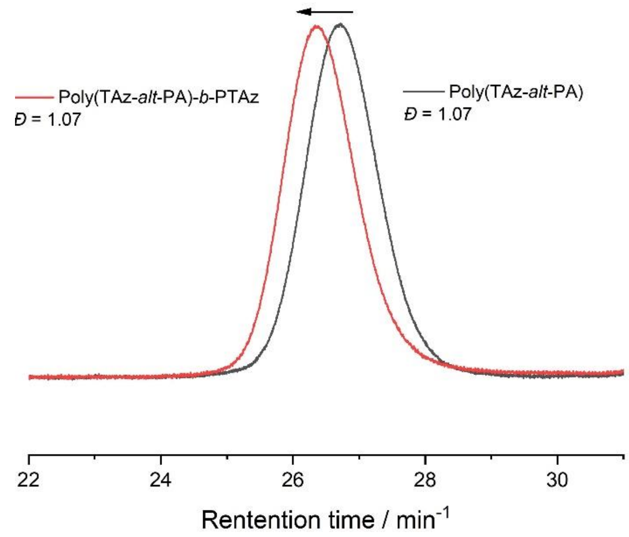

Figure S16. SEC traces of P(TAz-*alt*-PA) and P(PTAz-*alt*-PA)-*b*-PTAz diblock copolymers. (The black one in Entry 13)

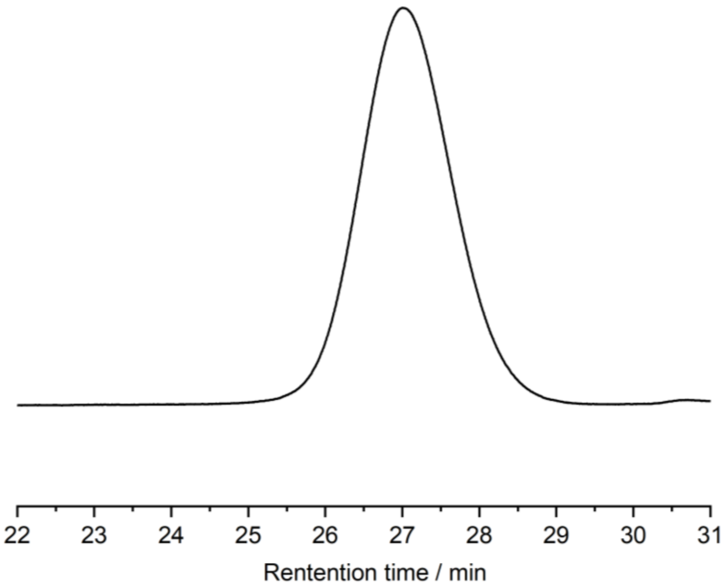

Figure S17. SEC trace of the copolymers of TAz and PA ($[TAz]_0/[PA]_0/[BA]_0/[t\text{-}BuP_2]_0$ = 15/15/1/0.3, 25°C, 24 h) (Entry 14).

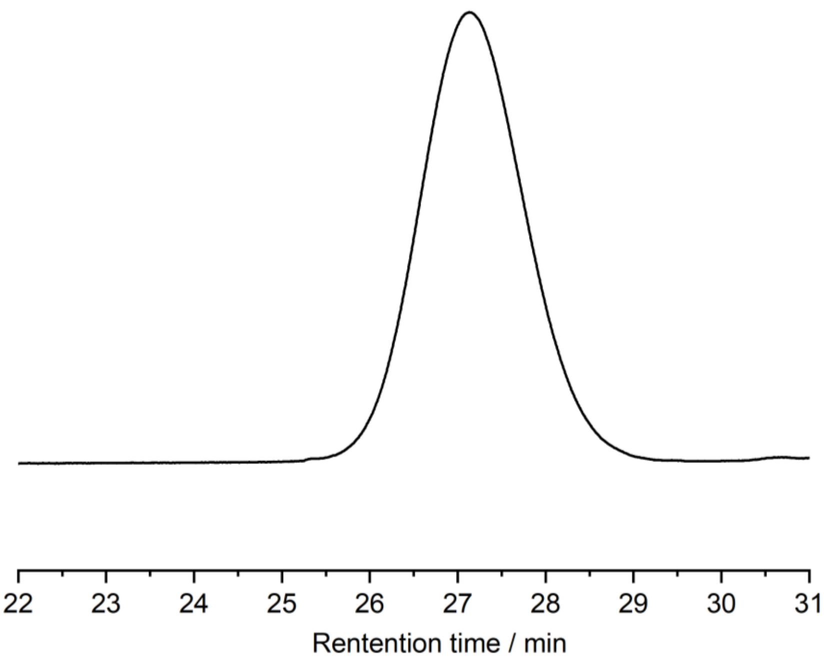

Figure S18. SEC trace of copolymers of TAz and PA ($[TAz]_0/[PA]_0/[BnOH]_0/[t\text{-}BuP_2]_0$ = 15/15/1/0.3, 25°C, 24 h) (Entry 15).

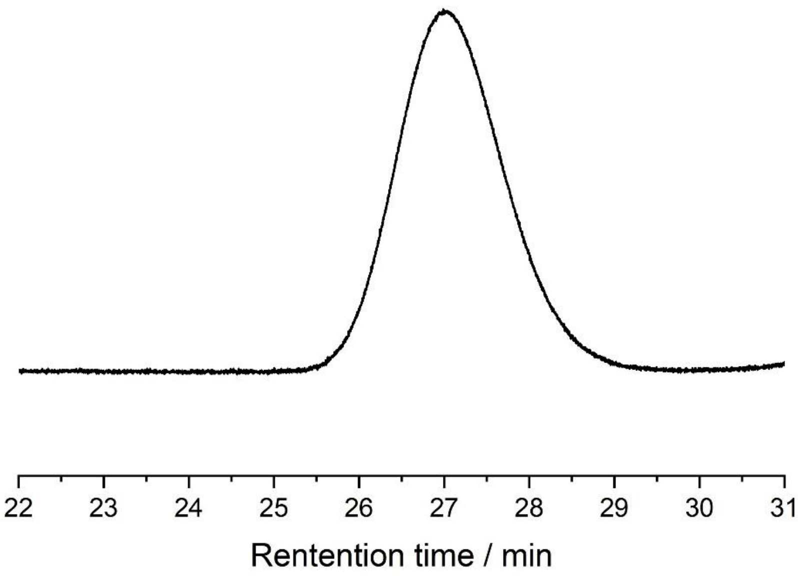

Figure S19. SEC trace of copolymers of TAz and PA ($[TAz]_0/[PA]_0/[1,4\text{-benzenedimethanol}]_0/[t\text{-}BuP_2]_0$ = 15/15/1/0.6, 25°C, 24 h) (Entry 16).

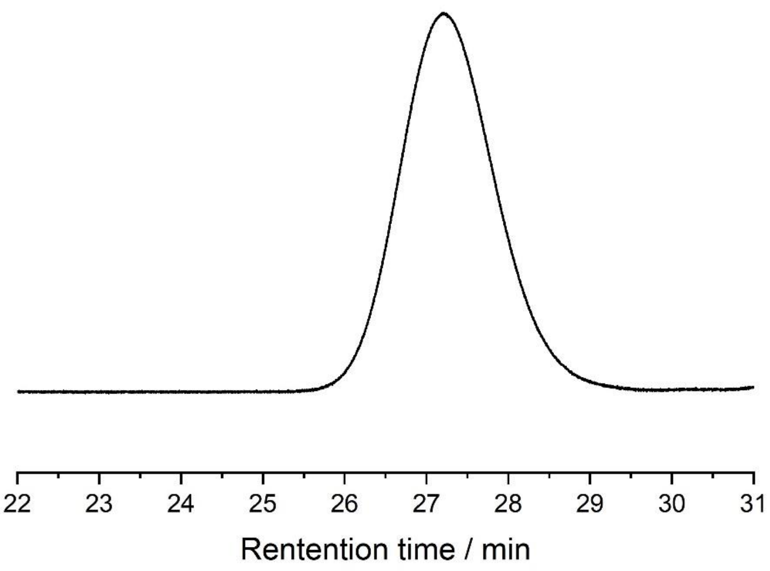

Figure S20. SEC trace of the copolymers of TAz and PA ($[TAz]_0/[PA]_0/[1,3,5\text{-benzenetrimethanol}]_0/[t\text{-}BuP_2]_0$ = 15/15/1/0.9, 25°C, 24 h) (Entry 17).

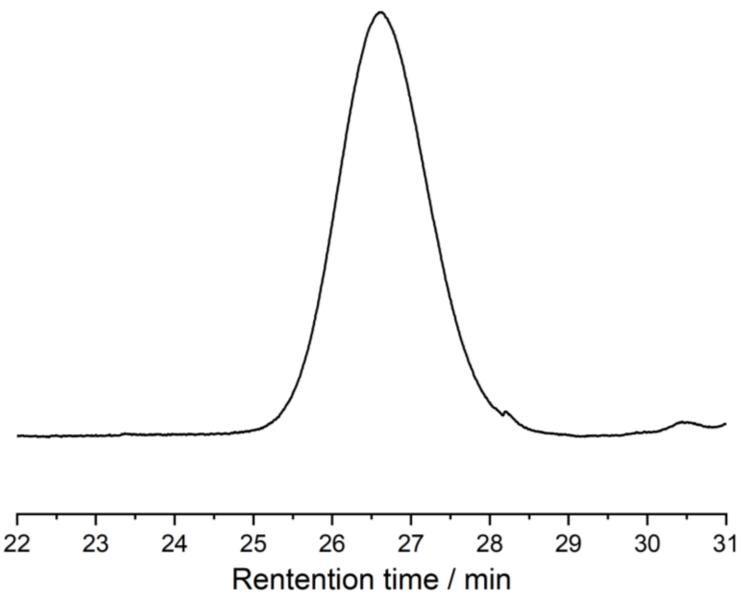

Figure S21. SEC trace of the copolymers of TAz and PA ($[TAz]_0/[PA]_0/[BnN(H)Ts]_0/[t\text{-}BuP_2]_0$ = 15/15/1/0.3, in DMF, 25°C, 24 h) (Entry 18).

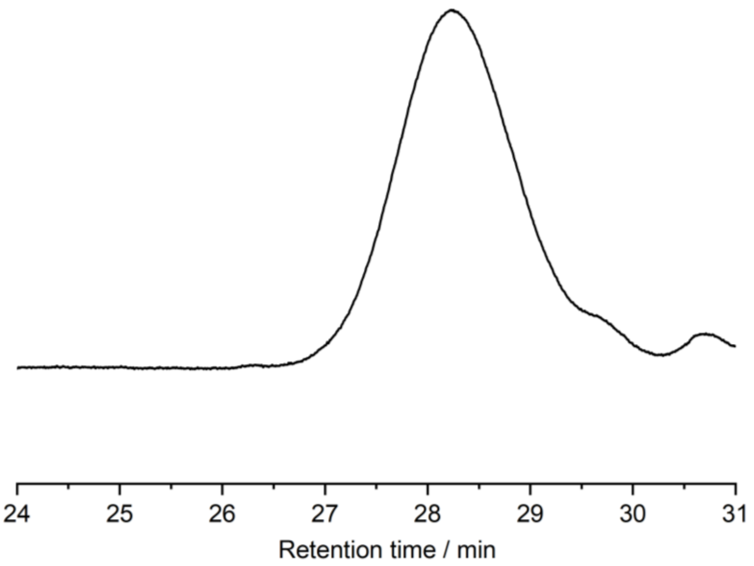

Figure S22. SEC trace of the copolymers of TAz and PA ($[TAz]_0/[PA]_0/[BnN(H)Ts]_0/[t\text{-}BuP_2]_0$ = 15/15/1/0.3, in CH_2Cl_2, 25°C, 24 h) (Entry 19).

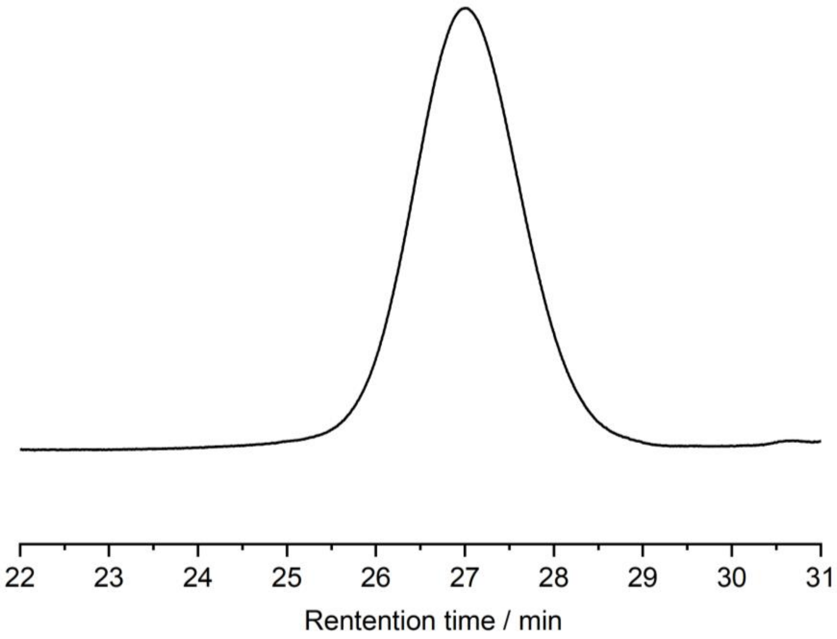

Figure S23. SEC trace of the copolymers of BAz and PA ($[BAz]_0/[PA]_0/[BnN(H)Ts]_0/[t\text{-}BuP_4]_0$ = 15/15/1/0.3, in THF, 25°C, 12 h) (Entry 20).

7. FTIR for copolymers

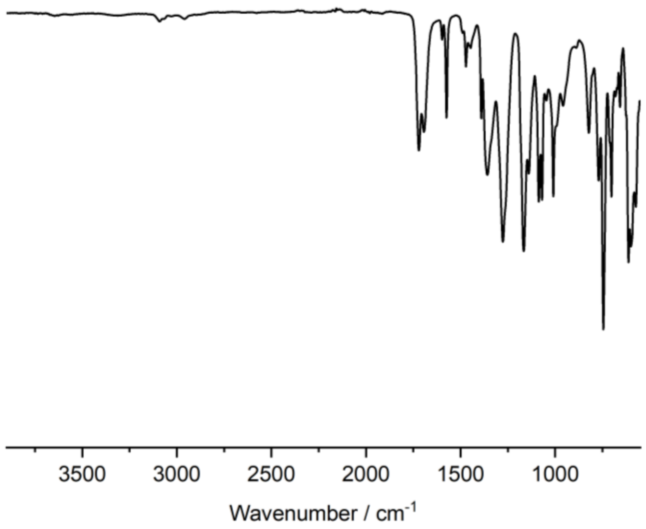

Figure S24. FTIR spectrum of the copolymer of BAz and PA.

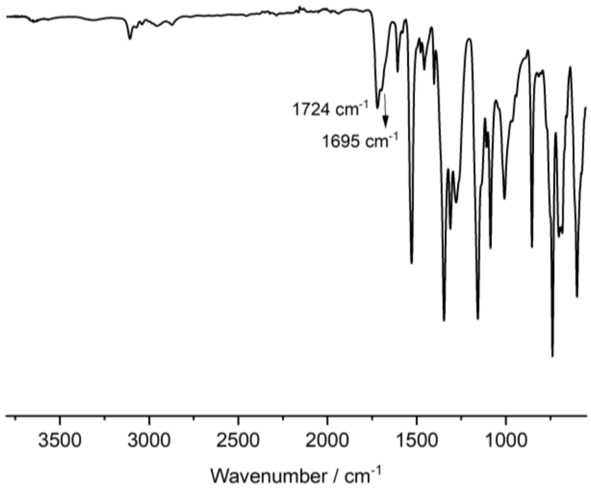

Figure S25. FTIR spectrum of the copolymer of NAz and PA.

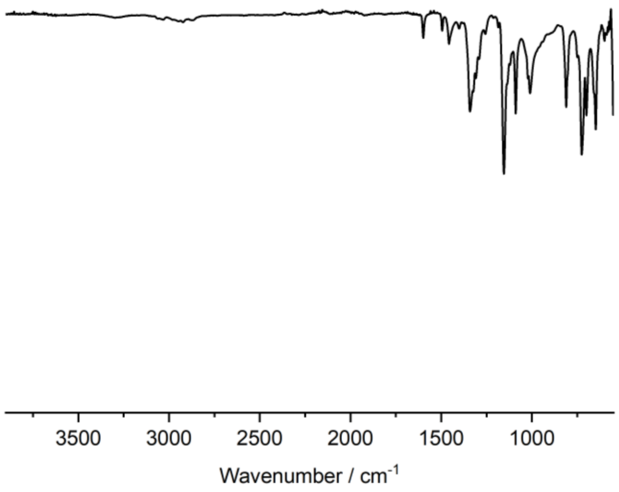

Figure S26. FTIR spectrum of PTAz

8. Kinetic study

Table S1. Experimental data for kinetic copolymerization experiments.

$[TAz]_0:[PA]_0:[I]_0:[t\text{-BuP}_2]_0$	$[PA]_{0,theor}$ / M	$[PA]_{0,exptl}$ / M	k_{obs}/ mol L^{-1} s^{-1}
18:15:1:0.3	1.0	1.11	3.37×10^{-5}
24:15:1:0.3	1.0	1.13	4.97×10^{-5}
30:15:1:0.3	1.0	1.17	6.00×10^{-5}
35:15:1:0.3	1.0	1.12	7.31×10^{-5}
40:15:1:0.3	1.0	1.02	9.369×10^{-5}
30:12:1:0.3	0.8	0.82	6.00×10^{-5}
30:9:1:0.3	0.6	0.54	6.29×10^{-5}
30:6:1:0.3	0.4	0.31	6.10×10^{-5}
30:15:1:0.5	1.0	1.08	1.38×10^{-4}
30:15:1:0.8	1.0	0.85	3.37×10^{-4}
30:15:1:1	1.0	0.53	5.28×10^{-4}

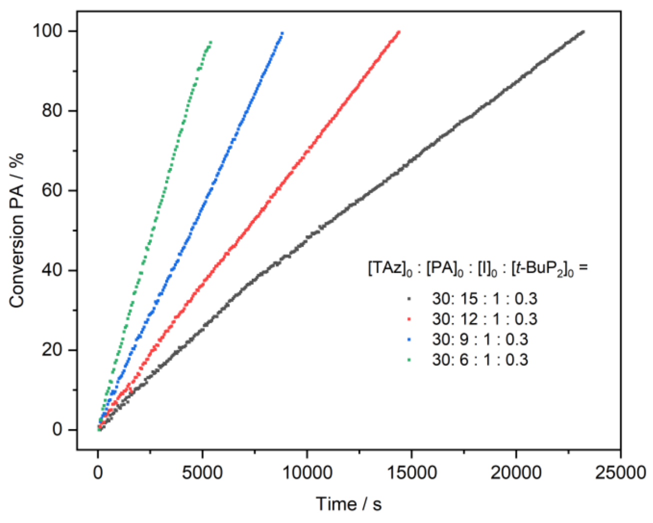

Figure S27. These reactions were performed by t-BuP$_2$ as a catalyst in THF ([TAz]$_0$ = 2 M) at 25°C with a different amount of PA. The automatic sampling interval of *in-situ* FTIR is 1 min before 8 h, 5min after 8 h.

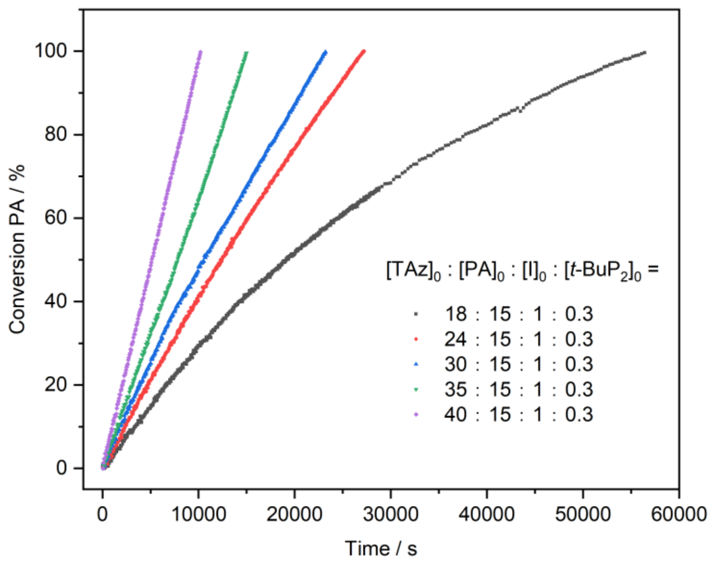

Figure S28. These reactions were performed by t-BuP$_2$ as a catalyst in THF ([PA]$_0$ = 1 M) at 25°C with a different amount of TAz. The automatic sampling interval of *in-situ* FTIR is 1 min before 8 h, 5 min after 8 h.

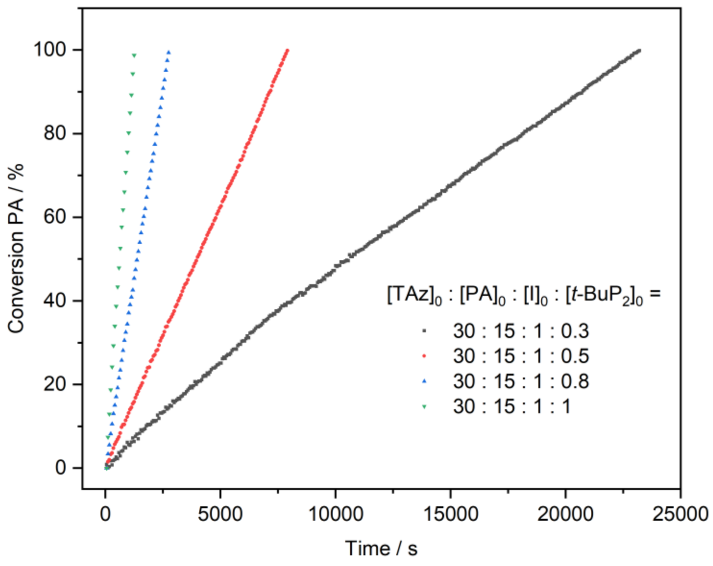

Figure S29. These reactions were performed by t-BuP_2 as a catalyst in THF ($[PA]_0$ = 1 M) at 25°C with a different amount of t-BuP_2. The automatic sampling interval of *in-situ* FTIR is 1 min before 8 h, 5 min after 8 h.

9. NMR titration for mechanism

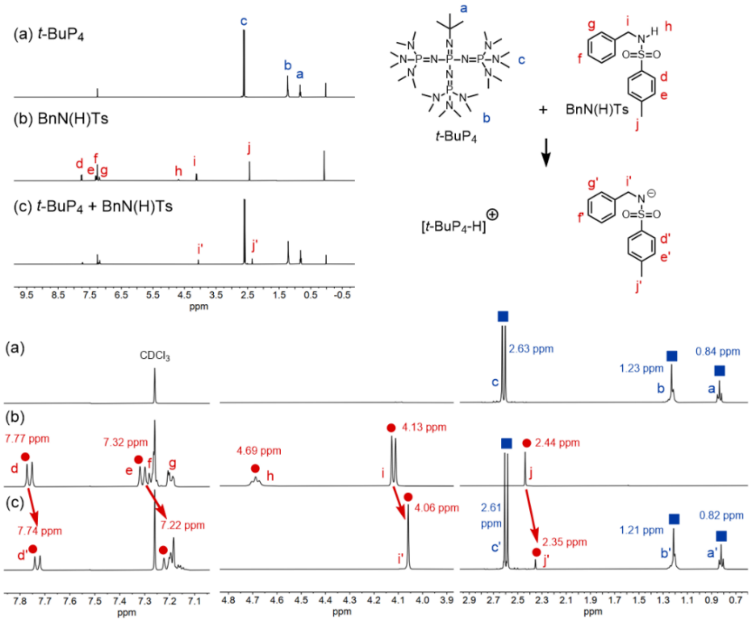

Figure S30. ^{1}H NMR (400 MHz, $CDCl_3$, 25°C) spectra of (a) t-BuP_4, (b) BnN(H)Ts, and (c) t-BuP_4 : BnN(H)Ts = 1 : 1 in $CDCl_3$ at same concentration of 0.08 mol L^{-1}.

10. NMR spectra of reactants

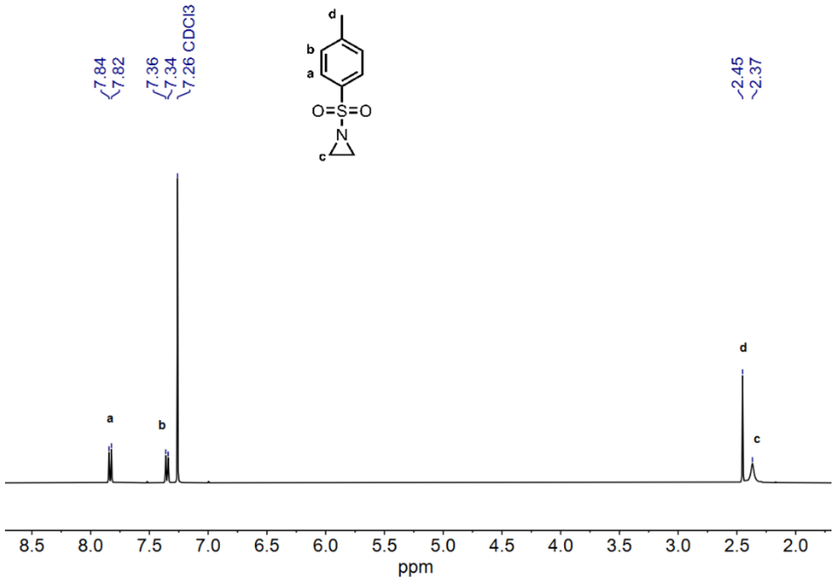

Figure S31. ^{1}H NMR (400 MHz, $CDCl_3$, 25°C) spectrum of TAz

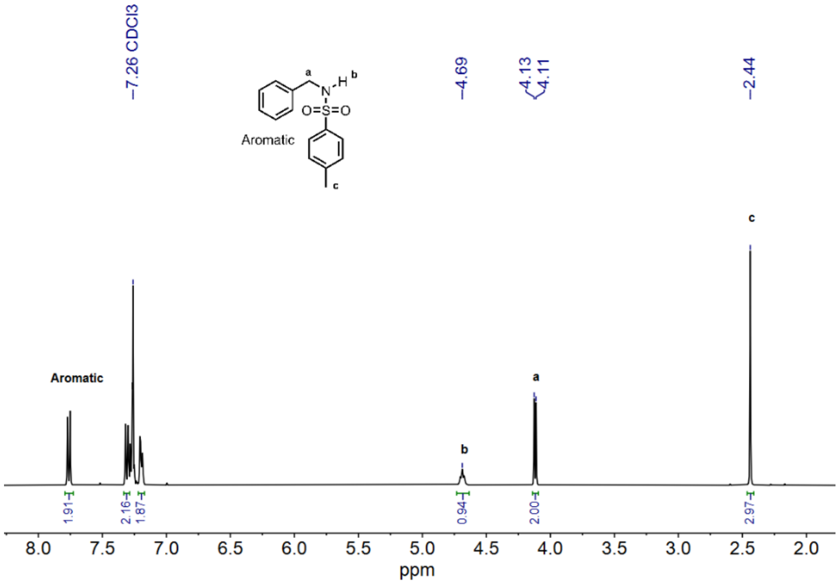

Figure S32. ^{1}H NMR (400 MHz, $CDCl_3$, 25°C) spectrum of BnN(H)Ts

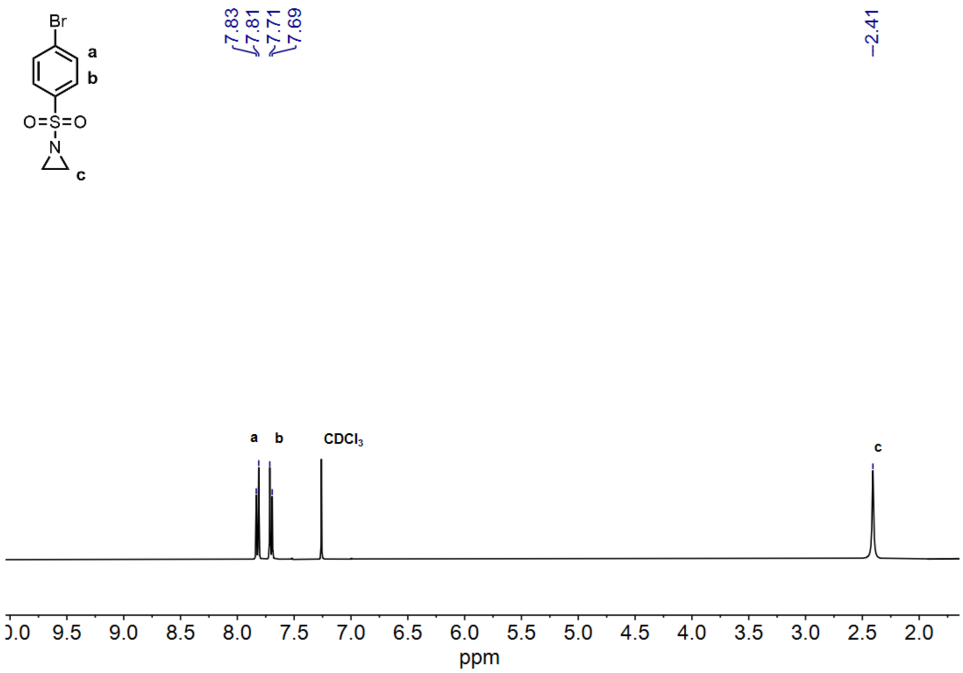

Figure S33. ^{1}H NMR (400 MHz, $CDCl_3$, 25°C) spectrum of BAz

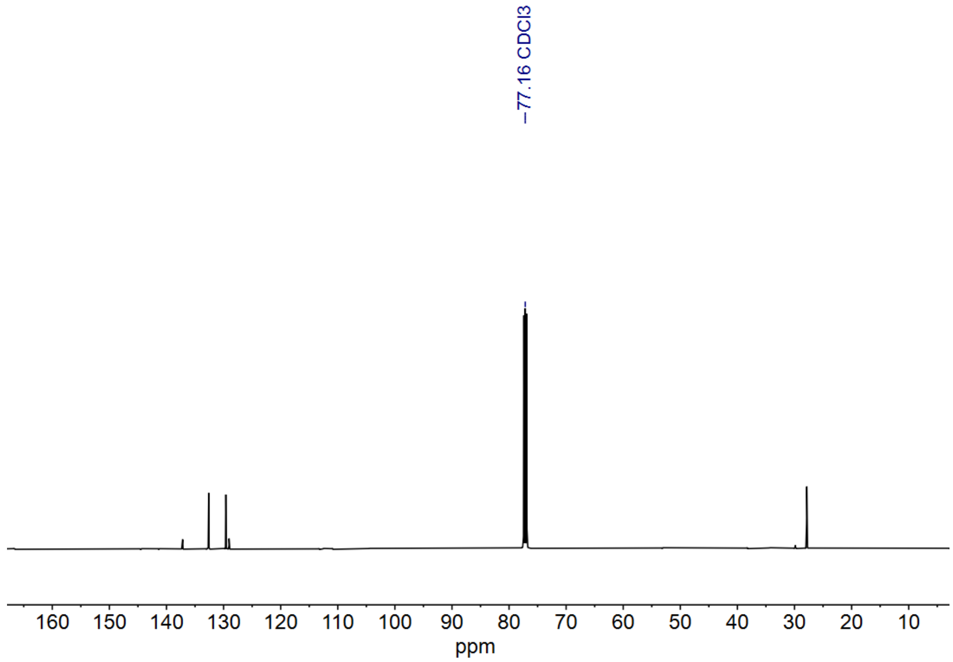

Figure S34. ^{13}C NMR (100 MHz, $CDCl_3$, 25°C) spectrum of BAz

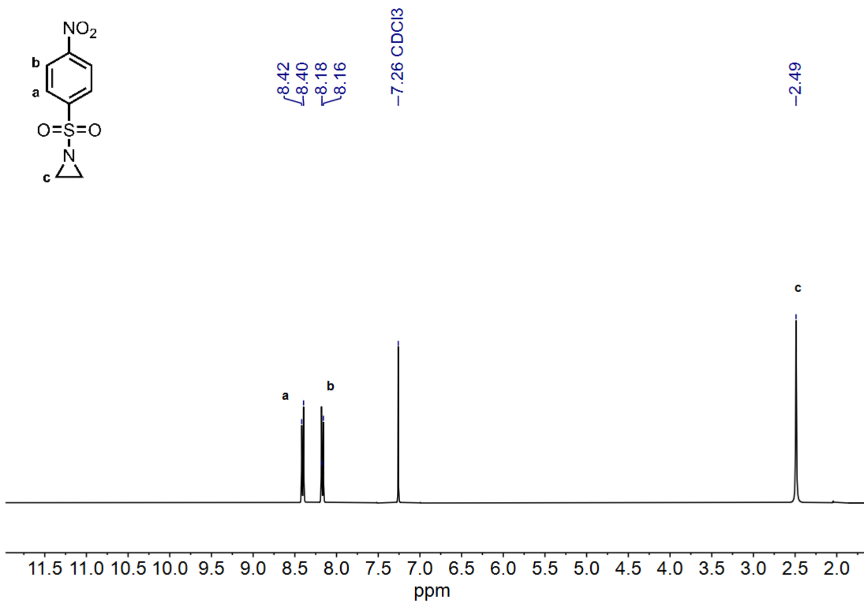

Figure S35. ^{1}H NMR (400 MHz, $CDCl_3$, 25°C) spectrum of NAz

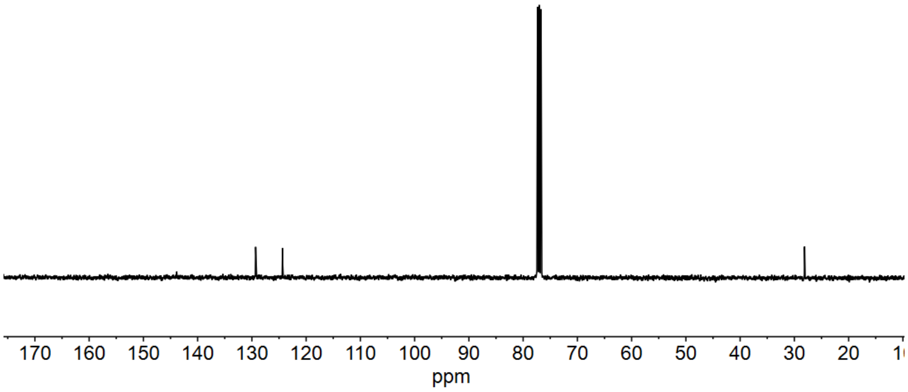

Figure S36. ^{13}C NMR (100 MHz, $CDCl_3$, 25°C) spectrum of NAz

[1] a) R. T. Skerlj, S. Nan, Y. Zhou, G. J. Bridger, *Tetrahedron Lett.* **2002**, *43*, 7569-7571; b) M. L. Duda, F. E. Michael, *J. Am. Chem. Soc.* **2013**, *135*, 18347-18349; c) F. Beltran, I. Fabre, I. Ciofini, L. Miesch, *Org. Lett.* **2017**, *19*, 5042-5045.
